# Vegetation Increases CH_4_ Emissions and Methanotroph Diversity in Marine Sediments

**DOI:** 10.1111/gcb.70989

**Published:** 2026-07-07

**Authors:** Marion Dolivet‐Maréchal, Carlos Palacin‐Lizarbe, Henri M. P. Siljanen, Dhiraj Paul, Abigaïl Delort, Jonathan Gervaix, Charline Creuzé des Châtelliers, Sabine Schmidt, Mathis Cognat, David Sebag, Olivier Taugourdeau, Clara Schübert, Nathalie Labourdette, Isabelle Bertrand, Lorenzo M. W. Rossi, Xavier Le Roux, Agnès Richaume, Alessandro Florio

**Affiliations:** ^1^ Laboratoire d'Ecologie Microbienne, UMR CNRS 5557, UMR INRAE 1418, VetAgro Sup Université Claude Bernard Lyon 1 Villeurbanne France; ^2^ Center for Advanced Studies of Blanes (CEAB‐CSIC) Blanes Spain; ^3^ Department of Environmental and Biological Sciences University of Eastern Finland Kuopio Finland; ^4^ Univ. Bordeaux, CNRS, Bordeaux INP, EPOC, UMR 5805 Pessac France; ^5^ Seaboost Montpellier France; ^6^ Earth Sciences and Environmental Technologies Division IFPEN Rueil‐Malmaison France; ^7^ Egis Group, Service Line Eau, Environnement et transition énergétique Montpellier France; ^8^ Univ. Bordeaux, CNRS, Bordeaux INP, EPOC‐OASU, UMR 5805 Station Marine d'Arcachon France; ^9^ UMR Eco&Sols, Univ Montpellier, CIRAD, INRAE, IRD, InstitutAgro Montpellier Montpellier France; ^10^ Department of Agricultural and Environmental Sciences University of Milan Milano Italy

**Keywords:** blue carbon, carbon cycling, functional genes (*mcrA*, *mmoX*, *pmoA*), greenhouse gas, methane cycling, methanogens and methanotrophs diversity, seagrass meadows, vegetated coastal environment, *Zostera noltii*

## Abstract

Seagrass meadows are key blue carbon (C) ecosystems, storing large amounts of organic C over centuries. Their climate benefits may be reduced by methane (CH_4_) emissions, whose microbial and environmental descriptors in *Zostera noltii* meadows, dominant seagrass in North‐Western Europe, remain poorly understood. We studied CH_4_ fluxes, CH_4_‐producing and consuming microbial communities and sediment physicochemical parameters in *Z. noltii* meadows and adjacent bare sediments across seven sites in Arcachon Bay, France. In situ CH_4_ fluxes were measured at low tide during daytime conditions, providing standardized estimates of peak emissions. Microbial communities were characterized using targeted metagenomics of three functional genes (*mcrA*, *mmoX*, *pmoA*) and quantitative PCR. CH_4_ fluxes were higher in vegetated than bare sediments (24.4 ± 2.6 vs. 9.4 ± 0.7 μmol m^−2^ day^−1^). Mixed linear models and random forest analyses identified C accumulation rate and CO_2_ flux as the strongest positive descriptors of CH_4_ fluxes. Vegetated sediments hosted more diverse methanotrophs, while methanogens showed no habitat differences. Four genera (*mcrA–Methanolobus*, *mmoX–Methylocella*, *pmoA–Methylococcus*, *Methyloglobulus*) emerged as abundant, seagrass‐associated, correlated with CH_4_ fluxes, and highlighted by models. Functional diversity, especially *pmoA* richness, was a stronger microbial descriptor of CH_4_ fluxes than gene abundance or a specific genus. Findings indicate *Z. noltii* meadows enhance C burial and CH_4_ emission, with methanotroph diversity potentially mitigating CH_4_ emissions. Our results provide the first integrated assessment of CH_4_ fluxes and their descriptors in *Z. noltii* meadows, based on limited temporal coverage capturing the daytime peak emission conditions, highlighting the intertwined nature of C burial and CH_4_ emissions and the need to account for both in blue C climate assessments.

## Graphical abstract

Seagrass meadows enhance carbon burial but also increase methane (CH_4_) emissions from marine sediments. The presence of seagrass modifies sediment properties, creating carbon‐rich environments characterized by higher carbon accumulation and CO_2_ fluxes, which stimulate CH_4_ production and lead to higher emissions. At the same time, seagrass and carbon‐enriched conditions reshape microbial communities by promoting a more diverse methanotrophic community, enhancing CH_4_ oxidation and partially reducing emissions. Despite this microbial buffering effect, CH_4_ fluxes remain overall positive, indicating that seagrass ecosystems act as net sources of methane while simultaneously storing carbon.

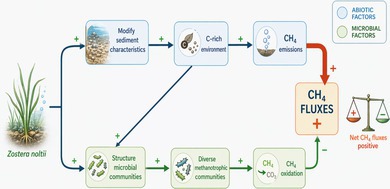

## 1 Introduction

Vegetated coastal ecosystems, such as seagrass meadows, play a critical role in global climate regulation by sequestering large amounts of organic carbon (C_org_) in their sediments over the long‐term. Despite occupying only ~0.1% of the ocean surface, seagrass meadows account for up to 18% of global ocean carbon (C) burial, primarily as C_org_ (Kennedy et al. 2010; McLeod et al. 2011). Thus, they are defined as key “blue carbon” habitats due to their high primary productivity and capacity to trap organic‐rich particles (Duarte et al. 2013; Fourqurean et al. 2012). Yet, the climate mitigation potential of seagrass meadows has recently been questioned due to several factors, including uncertainties and potential biases in C burial estimates, and/or their capacity to emit greenhouse gases (GHGs) including methane (CH_4_) (Bahlmann et al. 2015; Johannessen and Macdonald 2016; Garcias‐Bonet and Duarte 2017; Williamson and Gattuso 2022; Kristensen et al. 2025). As a potent GHG with a global warming potential 27 times higher than CO_2_, CH_4_ emissions from seagrass meadows can partially offset the climate benefit associated with C burial (IPCC 2023). Reported offsets vary widely across studies, ranging from negligible values (< 1%) to more than 10% depending on environmental conditions, species considered, and methodological approaches (Al‐Haj and Fulweiler 2020; Eyre et al. 2023; Dolivet‐Maréchal et al. 2026). However, global CH_4_ flux estimates from seagrasses remain highly uncertain due to the lack of knowledge on the abiotic and biotic determinants of these emissions (Tan et al. 2025; Yau et al. 2023). Within seagrass species, morphology and habitat can differ markedly, potentially influencing carbon burial and GHG emissions (Orth et al. 2006). For example, within the *Zostera* genus, *Zostera marina* is a larger, mostly subtidal species, whereas *Zostera noltii*, the focus of this study, is a temperate intertidal species with fine, dense rhizomes and smaller shoot biomass, forming either continuous meadows or patchy distributions (Den Hartog 1970; Auby 1991; Moore and Short 2006).

Methane emissions from seagrass sediments mainly arise from the balance between microbial production and consumption (Yau et al. 2023), which determines whether sediments are sources or sink of this GHG. Methanogens are mostly obligate anaerobic archaea and the primary CH_4_ producers in sediments (Conrad 2020; Schorn et al. 2022). These microorganisms use four main pathways, that is, hydrogenotrophic, acetoclastic, methylotrophic (Conrad 2020; Täumer et al. 2022), and methoxydotrophic methanogenesis, the latter being only recently described (Täumer et al. 2022). All pathways converge on the final step catalyzed by methyl‐coenzyme M reductase (MCR), encoded by the highly conserved *mcrA* gene, which remains a widely used biomarker to detect and quantify methanogens in environmental samples (Evans et al. 2019; Thauer 2019). Conversely, CH_4_ consumption in seagrass sediments is primarily driven by aerobic methanotrophs, which oxidize CH_4_ and thus substantially contribute to the CH_4_ sink (Laanbroek 2010; Liang et al. 2025). CH_4_ oxidation is catalyzed by methane monooxygenases (MMOs), enzymes existing in two forms: particulate (pMMO) and soluble (sMMO). The *pmoA* gene encodes a subunit of pMMO, while *mmoX* encodes a component of sMMO; both genes serve as reliable molecular markers for aerobic methanotrophic communities (Liang et al. 2025). The microbial mechanisms underpinning CH_4_ production and consumption in seagrass sediments are still poorly understood (Liang et al. 2025; Schorn et al. 2022). In sediments, recent studies indicate that methanogenic lineages are diverse, including *Methanosaetaceae*, *Methanosarcinaceae*, and *Methanobacteriales* (Chen et al. 2022; Schorn et al. 2022; Täumer et al. 2022) while aerobic methanotrophs are dominated by *Gamma‐* and *Alphaproteobacteria* (Liang et al. 2025; Täumer et al. 2022). However, most research has focused on a single functional gene (*mcrA*) or 16S rRNA gene‐based community analyses. Within the *Zostera* genus, a few studies on *Z. marina* and *Z. japonica* have quantified *mcrA* and used 16S rRNA sequencing to infer methanotrophic diversity, without directly targeting *pmoA* or *mmoX* (Liu et al. 2021; Tan et al. 2025). Thus, the full functional diversity of CH_4_‐cycling microbes in *Zostera* sediments remains unknown. To date, no study has simultaneously examined the abundance and diversity indices of the three key CH_4_‐cycling genes, *mcrA*, *mmoX*, and *pmoA* in *Z. noltii* sediments.

CH_4_ fluxes in seagrass ecosystems can vary widely across spatial and temporal scales (Maher et al. 2015; Rosentreter et al. 2021), yet there is no overarching general descriptor. Most studies tend to focus on either environmental descriptors, classically organic matter (OM) content and quality, which are often reported as key factors influencing CH_4_ fluxes (Al‐Haj and Fulweiler 2020), or microbial community dynamics (Schorn et al. 2022). Few studies integrate both environmental and microbial descriptors simultaneously (Tan et al. 2025). Additionally, comparative microbial analyses of vegetated and adjacent bare sediments remain scarce (Tan et al. 2025), limiting our understanding of seagrass‐specific microbial controls on CH_4_ fluxes. Furthermore, research has predominantly targeted large tropical seagrass species, whereas temperate dwarf species such as *Zostera noltii* , dominant in North‐Western Europe, remain poorly characterized in terms of both CH_4_ fluxes and microbial CH_4_‐cycling communities (Al‐Haj and Fulweiler 2020; Eyre et al. 2023).

In a recent study, in the Arcachon Bay, which hosts the largest *Z. noltii* population in Europe, we showed that CH_4_ fluxes were significantly higher in vegetated than in bare sediments (24.4 ± 2.6 vs. 9.4 ± 0.7 μmol m^−2^ day^−1^), with total emissions representing 6.1% ± 0.8% of local C burial (Dolivet‐Maréchal et al. 2026). Here, we aim to investigate why CH_4_ fluxes differ between vegetated and unvegetated sediments, how they are regulated by environmental descriptors, and how the presence of seagrass structures methanogenic and methanotrophic communities. We hypothesized that CH_4_ fluxes would be explained by the quantity (i.e., C_org_ %) and quality (i.e., lability) of OM stored in sediments, and that higher fluxes in seagrass meadows would be associated with greater diversity and abundance of methanogen‐related gene. Using an innovative targeted metagenomic approach and quantitative PCR, we screened for CH_4_‐related genes (*mcrA*, *mmoX*, *pmoA*) across 83 sediment samples, collected from both seagrass meadows and adjacent bare sediments. This scale of sampling is, to our knowledge, unprecedented for microbial studies in seagrass ecosystems. We combined these gene‐based microbial analyses with in situ CH_4_ flux measurements conducted during low tide, when gas exchange between the sediments and the atmosphere is maximized (Deborde et al. 2010), along with physicochemical, hydrological, and sedimentary parameters. Finally, we integrated all data using two types of modeling approaches, that is, mixed linear models (MLM) and random forest analysis (RFA), to identify key environmental descriptors of CH_4_‐cycling. This allowed us to better understand CH_4_ fluxes and to more accurately evaluate and predict the contribution of *Z. noltii* meadows to C budgets in temperate seagrass ecosystems.

## 2 Materials and Methods

### 2.1 Study Site and Habitat Characteristics

**FIGURE 1:**
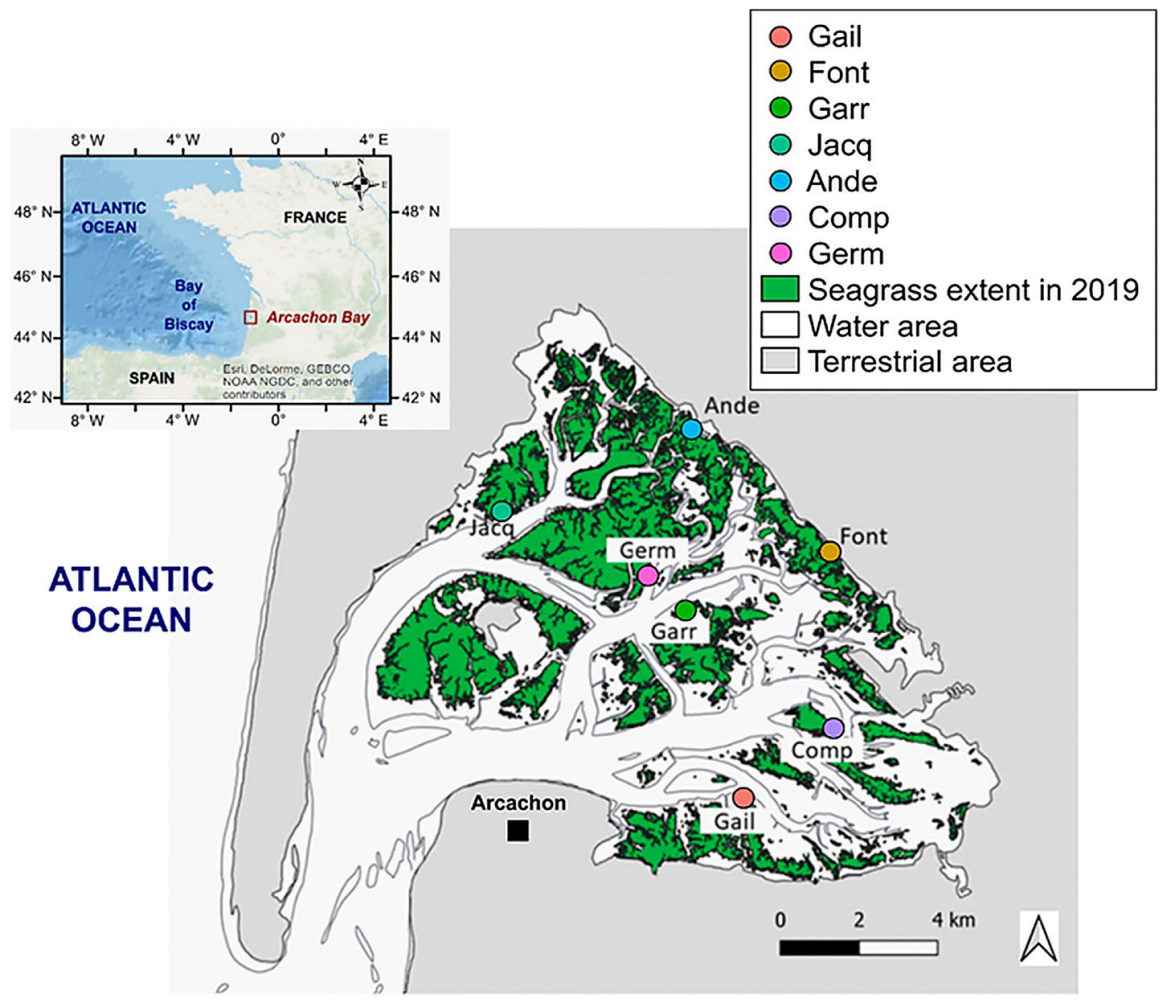
Location of Arcachon Bay and the seven studied sites with 2019 *Z. noltii* coverage. “Map lines delineate study areas and do not necessarily depict accepted national boundaries.”

Arcachon Bay is a temperate coastal lagoon located on the southern Atlantic coast of France (44°40′ N, 1°10′ W), covering an area of approximately 174 km^2^ (Figure 1). Subject to mesotidal to macrotidal regimes, the bay is connected to the open ocean via two main inlets and is characterized by semidiurnal tides that drive strong water exchange. In the Arcachon Bay, *Z. noltii* predominantly forms monospecific meadows in the sheltered intertidal mudflats, and represents the largest *Z. noltii* seagrass beds in Europe (Auby et al. 2011). Their abundance has declined by 44% during the last three decades (Muller et al. 2024) because of extreme weather events including heatwaves and storms (Auby et al. 2011; Gamain et al. 2018). As a result, sediment distribution across the bay is spatially heterogeneous: coarse sands and gravels dominate the main tidal channels, sandy muds accumulate in secondary channels, and finer silts are prevalent in intertidal zones (Cognat et al. 2018).

### 2.2 Experimental Design and Sampling Strategy

To encompass the environmental diversity of Arcachon Bay, seven sites were selected based on contrasting hydrodynamic regimes, sediment characteristics, and vegetation status (Figure 1). Each site included two adjacent areas: one vegetated by *Z. noltii* meadows (“Seagrass”) and a nearby unvegetated zone (“Bare”), hereafter referred to as subsites. Historical aerial imagery analyses indicated that these bare areas were vegetated prior to 2005, suggesting potential legacy C storage, with CH_4_ production persisting long after plant loss likely sustained by residual methylated compounds from decomposed seagrass material (Schorn et al. 2022). Both types of habitat have remained environmentally stable for over 20 years, providing a consistent framework for comparison.

Two field campaigns were conducted during the weeks commencing September 12, 2022, and August 28, 2023, that is, during the peak biomass of *Z. noltii* (Auby et al. 2011). The same sites and subsites were revisited in both years. In 2022, one sediment core per subsite was collected to quantify variables used to estimate C burial, including sediment accumulation rate (SAR), mass accumulation rate (MAR), carbon accumulation rate (CAR), and C_org_, using a PVC corer (7 cm internal diameter, 35 cm length), allowing the retrieval of approximately 20 cm of sediment. In 2023, GHG fluxes (CH_4_ and CO_2_) were measured in situ using opaque static flux chambers (details in Section 2.3), deployed once per day at midday and at low tide. At each subsite, a 5 m radius circular area was delineated to represent local habitat conditions. Within this area, six sampling points were randomly distributed and defined as replicates for GHG flux measurements. Immediately after each flux measurement, three sediment cores (10 cm depth) were collected at the exact chamber location and pooled into a single composite sample per replicate, representing the surface sediment layer commonly used for GHG and microbial analyses. A total of 83 flux measurements and composite sediment samples were analyzed across the bay (see Dolivet‐Maréchal et al. 2026 for more details). Each subsite included six replicates of flux measurements and associated sediment samples, except for Comp‐Bare (*n* = 5). To preserve in situ conditions and prevent microbial alteration, samples were stored in dark either at 4°C (for incubations and sediment characterizations) or −20°C (for molecular analyses). Sediment physicochemical parameters were quantified as described in Supporting Information.

### 2.3 In Situ and Laboratory GHG Measurements

CH_4_ and CO_2_ fluxes were measured in situ using a LI‐COR static opaque Smart Chamber system (model 8200‐01S) coupled with a trace gas analyzer (LI‐7810; LI‐COR Biosciences, USA). The chamber (volume 4244.1 cm^3^) was deployed on a PVC base (radius 10 cm, height 12 cm) previously inserted into the sediment. A 5‐min resting period was allowed to stabilize the sediment before measurements. Gas fluxes were then recorded over a 3‐min period. Data were recorded every second and processed using LI‐COR SoilFluxPro software. Flux rates were calculated based on the rate of gas concentration change over time, and expressed in μmol CH_4_ m^−2^ day^−1^ or μmol CO_2_ m^−2^ day^−1^ (details in Dolivet‐Maréchal et al. 2026), for comparability with the literature. Measurements were performed at midday during low tide on exposed sediments, when CH_4_ emissions are expected to be near‐maximum due to sediment oxygenation and temperature conditions (Deborde et al. 2010; Rosentreter et al. 2021). These daytime, low‐tide fluxes do not capture diel or tidal variability, and thus should be interpreted as representative of peak emissions rather than full 24‐h cycles.

To measure microbial activities, that is, anaerobic methanogenesis coupled to CO_2_ production, aerobic methanotrophy and aerobic CO_2_ production, 15 g of fresh sediment were transferred into transparent airtight flasks sealed with rubber stoppers and incubated at 28°C. Gas concentrations were measured using a gas chromatograph equipped with a micro‐catharometer detector (μGC‐R990; SRA instruments, Marcy L'Etoile, France). For methanogenesis and anaerobic CO_2_ production, the headspace was flushed with helium to establish anoxic conditions. CH_4_ and CO_2_ concentrations were measured every 4 h, starting 6 h after incubation began, over a 48‐h period. Methanotrophy was assessed using a newly developed protocol under aerobic conditions and with the headspace supplemented with 10% CH_4_. Methane concentration was measured every 6 h, starting 2 h after incubation, continuing for 74 h. These measurements provide an estimate of potential methanotrophic activity, allowing for quantitative comparison across samples, although they do not directly represent in situ rates. Such incubation‐based approaches are widely used to assess methane‐oxidation capacity in sediments with similar incubation conditions as we have used (Hanson and Hanson 1996; Reeburgh 2007). Aerobic CO_2_ production was measured every hour over 6 h. Microbial activity rates were estimated from the slope of the linear regression of CH_4_ (μg C‐CH_4_ g^−1^ dry soil h^−1^) or CO_2_ concentration (μg C‐CO_2_ g^−1^ dry soil h^−1^) over time and converted to μmol CH_4_ or CO_2_ m^−2^ day^−1^. The slope of the linear regression was calculated individually for each sample over the same time intervals, and all regressions showed a high goodness of fit (*R* ^2^ > 0.90).

### 2.4 Abundance of Bacteria 16S rRNA and Functional CH_4_‐Cycling Genes

The abundances of bacterial 16S rRNA and CH_4_‐cycling genes (*mcrA*, *mmoX*, *pmoAIa*, *pmoAIb*, and *pmoAII*) were estimated via quantitative real‐time PCR after DNA extraction and quantification from sediment samples (see full methodological details in Appendix S1: Materials and Methods and Table S1). Differences in primer coverage among functional genes should be considered, as *pmoA* was targeted using multiple primer sets whereas *mcrA* and *mmoX* were each amplified using a single primer set.

### 2.5 Targeted Metagenomic and Bioinformatic Pipeline

To investigate the functional diversity of CH_4_‐producing and consuming microorganisms, a targeted metagenomic approach was applied. Here, functional diversity was defined as the diversity of CH_4_‐cycling functional genes (*mcrA*, *mmoX*, *pmoA*) within each sediment sample, quantified using gene‐specific alpha diversity metrics as proxies. This method relied on sequence capture by probe hybridization to selectively enrich functional genes involved in CH_4_ and mineral nitrogen transformations, including *mcrA*, *mmoX*, and *pmoA*, as described in Putkinen et al. (2021) and Siljanen et al. (2025). Gene‐specific probes were designed based on clustered reference sequences at defined similarity thresholds (Siljanen et al. 2025). DNA sequencing was performed using the Illumina platform (Arbor Biosciences, Ann Arbor, MI, USA), producing approximately 5 million reads per sample. Raw sequences were processed using in‐house bioinformatic pipelines on the CSC Puhti supercomputer (Espoo, Finland). Gene sequences were identified using HMMER v3.3.2 via the nhmmer tool with a threshold of *E* < 0.0001 (Eddy 2022). Multiple sequence alignments were conducted with MAFFT (Katoh and Standley 2013), and phylogenetic placements were inferred with RAxML (version 8.2.12) (Stamatakis 2014). Community composition at the genus level was illustrated using ggplot2 in R (v4.4.0), after merging gene variants assigned to the same genus based on phylogenetic placement. Relative abundances were calculated as the proportion of gene sequences affiliated with each genus within a sample. These values were then multiplied by gene‐specific copy numbers per m^2^, as determined by qPCR, to estimate absolute abundances per m^2^. Genera detected for both *mmoX* and *pmoA* genes were labeled with “_m” for *mmoX* and “_p” for *pmoA* to distinguish between the two.

Alpha diversity metrics were calculated separately for each sample to assess the diversity of *mcrA*, *mmoX*, and *pmoA* gene sequences. Richness (number of unique placements) and Shannon diversity index were computed using the nplace count per phylogenetic placement as a proxy for abundance. These calculations were performed using the R package vegan (v1.44.0) (Oksanen et al. 2020), applying the specnumber() and diversity() functions to gene‐specific datasets. In this article, gene‐specific richness and diversity are referred to as gene_R and gene_D, respectively (e.g., mcrA_R, pmoA_D).

All sequences are available in the NCBI Sequence Read Archive (PRJNA1261814).

### 2.6 Mixed Linear Models and Random Forest Analysis

To assess the environmental and microbial descriptors of CH_4_ fluxes, MLM were employed. All explanatory variables were log_10_‐ or square root‐transformed to reduce the impact of extreme values and then scaled (*z*‐scores) to allow direct comparison of their relative influence (coefficient of the model). Model selection was performed using the dredge() function from the *MuMIn* R package, generating all possible combinations of descriptors within each predefined subset of variables (Table S2a–i). Then, to avoid overfitting, only the models with all significant descriptors (*p* < 0.05) were retained. Given the sample size and to avoid overfitting, ensure robust parameter estimation, and improve the ecological interpretability, the number of explanatory variables included in each model was limited to a maximum of five descriptors. To avoid multicollinearity, related variables (e.g., CAR and MAR) were not included in the same model.

Model performance was primarily evaluated using the corrected Akaike information criterion (AICc). Linear models were fitted using the lm() function, and mixed‐effects models were fitted using the lme() function in R (v4.3.1). For fixed‐effect models, generalized least squares (GLS) models were also fitted to allow direct comparison of AICc values with mixed‐effects models.

The Subsite random effect represented the combination of Site and Type (i.e., bare vs. vegetated) without a hierarchy, thus accounting simultaneously for variability among Sites and between Types; while we also build models with Site nested in Type (e.g., model 1, Table 3). Random effects were specified as random intercepts, allowing the baseline CH_4_ flux to vary among Sites, Types or Subsites. In addition, random slopes were tested for key variables to capture potential Site‐specific differences in the relationships between descriptors and CH_4_ fluxes, with slopes retained only when they improved model fit based on AICc, for example, as in Model 15 (Table 3). When competing models showed similar support (ΔAICc ≤ 2), likelihood ratio tests (ANOVA) were used to compare models and determine whether simpler models with fewer explanatory variables provided an equally adequate fit, in which case the most parsimonious model was retained. These models are described in Section 3.

Separate models were built for each subset of descriptors grouping them by categories, for example, physical, gas‐related, chemical and microbial; keeping key descriptors within each category (Table S2a–i, as in Palacin‐Lizarbe et al. 2020). The most significant descriptors selected from each subset‐specific model were then merged to create a combined subset (Table S2m) used to construct the final models. This two‐step approach was necessary because of the limited computational power of the dredge() function being able to test only up to 30 explanatory variables simultaneously; the subsetting ensured keeping informative descriptors from each subset in the final combined models. For the *mcrA* genus subset, this constraint also guided genus selection. Genera retained met at least one of the following criteria: (i) belonging to the five most abundant genera based on genus copy numbers per m^2^ across the Arcachon Bay, and/or (ii) being significantly correlated with in situ CH_4_ fluxes (*p* < 0.05). Because some genera fulfilled both criteria, the *mcrA* genus subset retained for modeling comprised 14 genera. We also tested an alternative configuration of the combined dataset, replacing *Methanopyrus* with *Methanosarcina*, to assess the robustness of the model under the same 30‐variable constraint. This modification did not affect the overall results or the main patterns observed.

In addition to MLM, we applied RFA as a complementary, nonparametric, ensemble‐based modeling approach using the same subsets of descriptors. While RFA does not explicitly account for random effects (e.g., Site, Type, or Subsite), it is well suited to capture complex and nonlinear relationships between explanatory variables and the response variable. This combined approach allowed us to cross‐validate the key descriptors identified by MLM and to assess the relative importance of variables without relying on assumptions of linearity or normality. Model tuning was performed using the tuneRF() function in R, testing different *mtry* values (i.e., the number of variables randomly sampled at each split) across 1000 trees. At each iteration, *mtry* was adjusted by a factor of 1.5, and the search continued only if the relative improvement in out‐of‐bag (OOB) error exceeded 1%. This procedure enabled the identification of the optimal *mtry* value while minimizing overfitting. By evaluating the mean increase in mean squared error (%IncMSE), key variables within each subset were highlighted, reinforcing the significance of descriptors previously identified in the MLM.

### 2.7 Statistical Analyses

All data analyses were conducted using R (version 4.4.0). Results are reported as means ± standard error (SE). Transformations (log‐, square‐root‐, or others) were applied when necessary to meet analysis assumptions, which were assessed using Shapiro–Wilk and Bartlett tests for normality and homogeneity of variances, respectively. However, as not all variables met these assumptions even after transformation, nonparametric tests were consistently applied. Differences between bare and vegetated sediments were tested by site and across the entire bay using Wilcoxon tests. To compare gene abundance, richness, and diversity across sites, Kruskal–Wallis tests were performed, followed by nonparametric post hoc pairwise comparisons using Dunn's test, with Benjamini–Hochberg correction for multiple testing. Spearman's rank correlation test was used to examine relationships between CH_4_ fluxes, production and oxidation rates.

Principal component analysis (PCA) was performed to explore patterns among physical, chemical, microbial, and GHG variables across all sampling sites, using the FactoMineR package in R. Additionally, a PCA based on Hellinger distance (Legendre and Gallagher 2001) was carried out to assess the ordination of microbial gene variants involved in CH_4_‐cycling. This analysis aimed to identify genera with gene variants that significantly influence the PCs. Significant correlations between variables and components were identified using the envfit() from the vegan package and were incorporated into PCA visualizations.

## 3 Results

### 3.1 Spatial Gradients Driving Sediment Functioning

**FIGURE 2:**
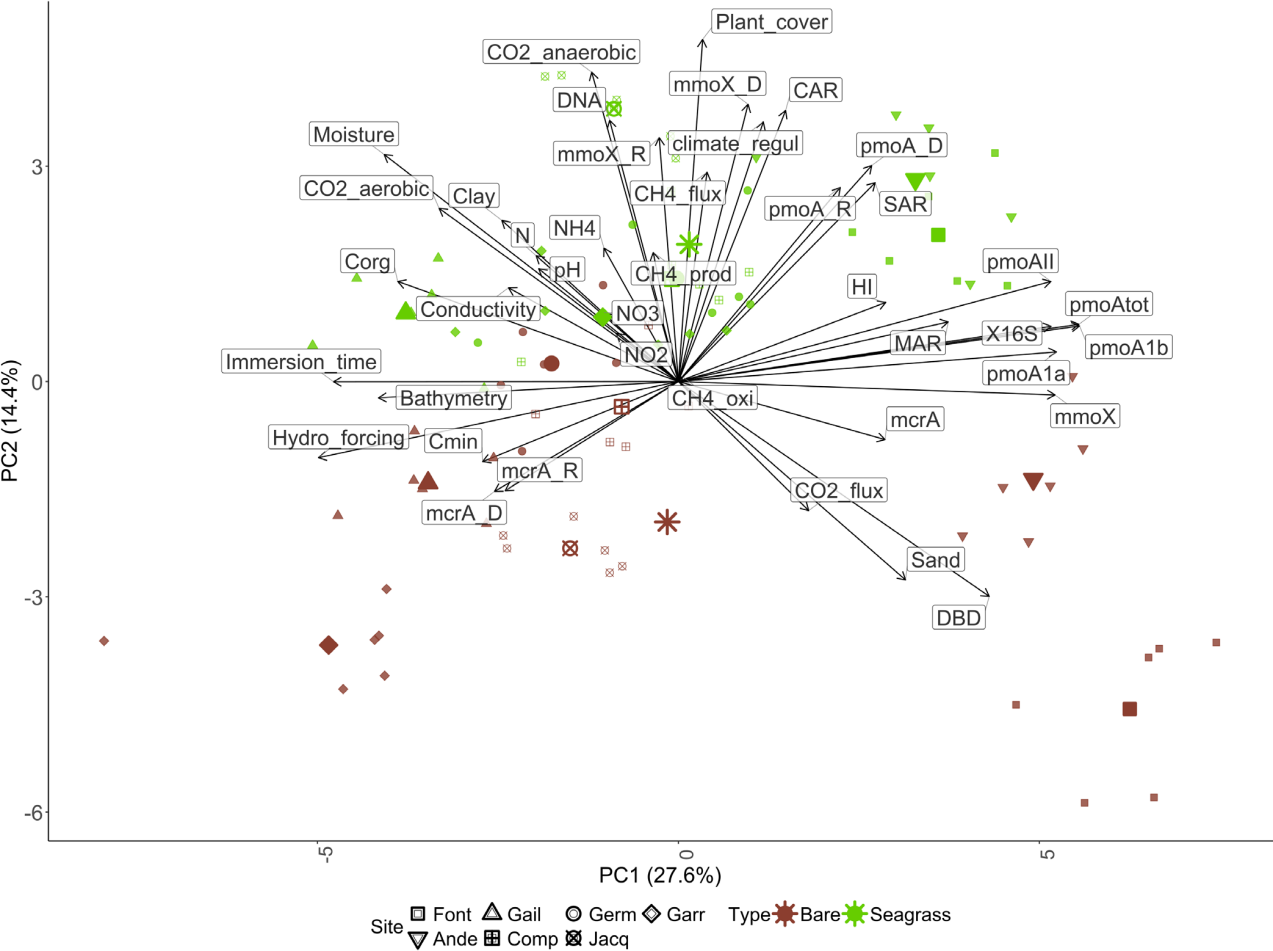
PCA biplot illustrating the relationships among physical, chemical, microbial, and GHG variables across all sampling sites. Arrows represent the contribution of each variable to the ordination. Variables include physical (e.g., sediment properties), chemical (e.g., nutrients, organic matter), microbial (e.g., functional genes), and GHG‐related descriptors. Both axes were rescaled (×6) to improve visualization of sites and variables. Sites are represented by different shapes. Color symbols denote the sediment Type (brown = Bare, green = Seagrass), *n* = 83. Larger symbols represent the barycenter of the Subsite, that is, the combination of Site and Type.

Two main gradients sorted the studied sediments. The first corresponded to a large‐scale spatial gradient, while the second was associated with the presence or absence of seagrass at a smaller spatial scale. The large‐scale gradient was linked to the connection to the ocean and to the sediment stability, as it was evidenced in the Principal Component 1 (PC1, Figure 2, Figure S1). On the left side of the PC1 are sorted sites that are more exposed to the hydrodynamic forcing, located near major tidal channels, and closer to the ocean inlet, characterized by longer immersion times, stronger current flows, and greater depths (higher bathymetry). These exposed sites are in more dynamic and physically constrained environments, where organic and mineral C arrive after being carried there by hydrodynamic forces. Conversely, on the right side of PC1 are the sites in the inner part of the bay. These sheltered sites have a lower degree of connection to the ocean and are characterized by calmer conditions, higher levels of labile C, and increased microbial abundance (Figure S1, Table S3).

The second main ecological gradient is driven by the presence of vegetation within sites. Bare sediments are on the negative side of PC2 while vegetated in the positive side (Figure 2). Most of the measured variables tend to have higher values in seagrass‐covered areas compared to bare sediments, with the exception of hydrodynamic forcing (Tables 1 and 2, Table S4). Vegetated subsites are characterized by higher clay content, greater sediment moisture, and elevated values of several biogeochemical variables, including: CH_4_ flux, aerobic and anaerobic CO_2_ production, CH_4_ production, SAR, MAR, CAR, climate regulation, nitrogen (N), ammonium (NH_4_^+^), and nitrate (NO_3_^−^) content (Tables 1 and 2, Table S4). On average across the bay, daytime CO_2_ fluxes measured at low tide were negative, indicating net uptake, with no consistent effect of vegetation. However, considerable variability was observed among sites, with two out of seven showing net positive fluxes (Table 1).

**TABLE 1:** GHG‐related variables across study sites, grouped by sediment Type (bare vs. seagrass). Sites are ordered by increasing CH_4_ fluxes measured in seagrass sediments. Values are mean ± SE. Within each Site, a Subsite (5 m radius) was defined for each sediment Type, and represents the mean of six replicate measurements. “All” values correspond to the mean of all individual measurements within each sediment Type (*n* = 41 bare, *n* = 42 seagrass). Data are hierarchical, with measurements nested within Subsites and Subsites nested within Sites. CO_2_ measurements are expressed in mmol m^−2^ day^−1^ and CH_4_ measurements in μmol m^−2^ day^−1^. Values of 0 indicate no detectable measurement (below detection resolution).

Site_ID	Type (subsite)	CH_4_ flux	CO_2_ flux	CO_2 aerobic_	CO_2 anaerobic_	CH_4_ production	CH_4_ oxidation
		μmol CH_4_ m^−2^ day^−1^	mmol CO_2_ m^−2^ day^−1^	mmol CO_2_ m^−2^ day^−1^	mmol CO_2_ m^−2^ day^−1^	μmol CH_4_ m^−2^ day^−1^	μmol CH_4_ m^−2^ day^−1^
Comp	Bare	10.8^NS^ ± 2.2	−55.0 ± 8.3	400.0 ± 59.0	170.0 ± 18.0	0.51 ± 0.06	0^NS^
	Seagrass	13.9^NS^ ± 1.5	−24.0** ± 4.5	660.0^#^ ± 79.0	390.0** ± 65.0	4.73^#^ ± 1.73	64.03^NS^ ± 64.03
Jacq	Bare	13.3^NS^ ± 1.1	−16.0** ± 1.8	510.0 ± 73.0	150.0 ± 9.4	0.93^NS^ ± 0.31	0^NA^
	Seagrass	14.5^NS^ ± 1.1	−53.0 ± 6.2	870.0** ± 57.0	290.0** ± 17.0	0.57^NS^ ± 0.36	0^NA^
Germ	Bare	9.0 ± 2.0	−24.0^NS^ ± 7.1	370.0 ± 19.0	130.0 ± 11.0	1.76^NS^ ± 0.67	0^NS^
	Seagrass	15.0^#^ ± 2.2	−22.0^NS^ ± 7.8	640.0** ± 76.0	230.0** ± 17.0	0.30^NS^ ± 0.08	17.10^NS^ ± 17.10
Font	Bare	8.1 ± 0.8	15.0^NS^ ± 1.0	99.0 ± 14.0	81.0 ± 7.9	0.03 ± 0.01	0^NS^
	Seagrass	27.0** ± 4.7	12.0^NS^ ± 3.2	560.0** ± 51.0	290.0** ± 44.4	3.49** ± 1.36	66.37^NS^ ± 49.39
Garr	Bare	11.4 ± 2.2	0.9^NS^ ± 2.6	880.0^#^ ± 40.0	160.0 ± 10.0	0.95 ± 0.18	81.71^NS^ ± 81.71
	Seagrass	29.7* ± 9.8	6.7^NS^ ± 2.0	720.0 ± 81.0	280.0** ± 22.0	2.34** ± 0.41	0^NS^
Gail	Bare	4.2 ± 0.2	−32.0^NS^ ± 5.8	530.0^NS^ ± 35.0	140.0^NS^ ± 19.0	0.63^NS^ ± 0.29	0^NA^
	Seagrass	35.0** ± 10.0	−25.0^NS^ ± 3.3	520.0^NS^ ± 83.0	190.0^NS^ ± 27.0	2.47^NS^ ± 1.42	0^NA^
Ande	Bare	9.0 ± 2.3	−22.0 ± 3.6	260.0 ± 25.0	110.0 ± 7.8	0.61^NS^ ± 0.42	55.15^NS^ ± 48.67
	Seagrass	35.9** ± 6.2	−6.1* ± 3.2	470.0* ± 76.0	200.0** ± 20.0	1.53^NS^ ± 0.55	31.51^NS^ ± 31.51
All	Bare	9.4 ± 0.7	−18.0^NS^ ± 3.6	440.0 ± 39.0	130.0 ± 6.2	0.78 ± 0.15	20.03^NS^ ± 13.82
	Seagrass	24.4*** ± 2.6	−16.0^NS^ ± 3.6	640.0*** ± 32.0	270.0*** ± 16.0	2.20* ± 0.43	25.57^NS^ ± 12.43

*Note:* Significant differences between bare and vegetated sediments were assessed using Wilcoxon tests: ^#^ *p* < 0.1, *p < 0.05, **p < 0.01, ***p < 0.001, NS = not significant.

**TABLE 2:** Microbial data for the studied sites, grouped by sediment Type (bare vs. seagrass). Values are reported as mean ± SE. Within each Site and sediment Type (bare or seagrass), replicate Subsites (5 m radius) were sampled at spatially distinct locations and represent the mean of 5–6 replicate measurements. “All” values correspond to the mean of all individual measurements within each sediment Type (bare: *n* = 41; seagrass: *n* = 42).

Site_ID	Type (subsite)	DNA	16S rRNA	mcrA	mmoX	pmoA1a	pmoA1b	pmoAII	pmoAtot	mcrA_R	mcrA_D	mmoX_R	mmoX_D	pmoA_R	pmoA_D
		ng m^−2^	copies m^−2^	copies m^−2^	copies m^−2^	copies m^−2^	copies m^−2^	copies m^−2^	copies m^−2^						
Comp	Bare	5.1e+08 ± 9.9e+07	1.4e+14 ± 1.1e+13	6.3e+09 ± 1.2e+09	1.3e+10^NS^ ± 1.6e+09	7.7e+10^NS^ ± 1.1e+10	7.6e+11^NS^ ± 9.7e+10	6.3e+10^NS^ ± 1.2e+10	9.0e+11^NS^ ± 1.2e+11	25.8^NS^ ± 4.3	2.8^NS^ ± 0.1	37.2^NS^ ± 1.8	3.1^NS^ ± 0.1	10.4^NS^ ± 1.5	0.4 ± 0.0
	Seagrass	1.2e+09** ± 1.8e+08	2.0e+14^#^ ± 1.9e+13	2.1e+10^#^ ± 6.8e+09	1.1e+10^NS^ ± 1.7e+09	6.5e+10^NS^ ± 9.2e+09	9.2e+11^NS^ ± 1.2e+11	7.6e+10^NS^ ± 1.4e+10	1.1e+12^NS^ ± 1.4e+11	27.3^NS^ ± 1.8	2.8^NS^ ± 0.1	33.8^NS^ ± 1.7	3.1^NS^ ± 0.0	8.8^NS^ ± 0.9	0.6^#^ ± 0.1
Jacq	Bare	3.8e+08 ± 8.7e+07	9e+13^NS^ ± 9.4e+12	1.5e+10^#^ ± 3.4e+09	6.8e+09^NS^ ± 3.6e+08	3.8e+10^NS^ ± 3.5e+09	5.1e+11 ± 3.9e+10	3.4e+10^NS^ ± 1.9e+09	5.8e+11 ± 4.3e+10	57.5^NS^ ± 18.5	3.1^NS^ ± 0.3	23.7 ± 1.4	2.4 ± 0.0	7.3^NS^ ± 1.4	0.3^NS^ ± 0.1
	Seagrass	2.9e+09** ± 6.9e+08	1e+14^NS^ ± 1.5e+13	7.0e+09 ± 1.6e+09	7.6e+09^NS^ ± 1.3e+09	4.3e+10^NS^ ± 5.2e+09	6.7e+11^#^ ± 6.0e+10	4.7e+10^NS^ ± 6.8e+09	7.6e+11* ± 7.1e+10	20.7^NS^ ± 2.4	2.7^NS^ ± 0.1	32.3* ± 1.3	3.0** ± 0.0	6.2^NS^ ± 0.9	0.5^NS^ ± 0.1
Germ	Bare	1.4e+09^NS^ ± 2.5e+08	7.9e+13 ± 1.0e+13	9.3e+09 ± 1.0e+09	9.8e+09 ± 1.5e+09	5e+10 ± 4.9e+09	6.6e+11 ± 6.4e+10	4.6e+10 ± 6.0e+09	7.5e+11 ± 7.3e+10	27.5^NS^ ± 3.0	2.9* ± 0.1	28.8^NS^ ± 2.8	2.9^NS^ ± 0.1	7.5^NS^ ± 0.7	0.5^NS^ ± 0.1
	Seagrass	1.6e+09^NS^ ± 2.4e+08	1.9e+14* ± 3.5e+13	2.3e+10* ± 4.8e+09	1.7e+10* ± 2.8e+09	1e+11^#^ ± 1.9e+10	1.2e+12^#^ ± 2.1e+11	9.7e+10* ± 1.5e+10	1.4e+12^#^ ± 2.4e+11	22.3^NS^ ± 2.5	2.6 ± 0.0	30.7^NS^ ± 2.0	3.0^NS^ ± 0.1	7.0^NS^ ± 0.7	0.6^NS^ ± 0.1
Font	Bare	5.6e+08 ± 5.7e+07	3.4e+14^NS^ ± 6.1e+13	2.3e+10 ± 9.6e+09	3.2e+10** ± 3.5e+09	1.5e+11^#^ ± 2.0e+10	2.0e+12^NS^ ± 3.1e+11	1.3e+11^NS^ ± 2.8e+10	2.3e+12^NS^ ± 3.5e+11	21.2 ± 2.6	2.7^NS^ ± 0.1	20.3 ± 2.6	2.6 ± 0.1	4.7 ± 1.3	0.5 ± 0.2
	Seagrass	7.6e+08^#^ ± 7.7e+07	2.7e+14^NS^ ± 3.6e+13	4.5e+10^#^ ± 4.9e+09	1.5e+10 ± 2.8e+09	9.7e+10 ± 1.9e+10	1.5e+12^NS^ ± 1.3e+11	9.8e+10^NS^ ± 1.4e+10	1.7e+12^NS^ ± 1.6e+11	34.3^#^ ± 4.3	2.7^NS^ ± 0.1	29.3* ± 1.9	3.0* ± 0.1	15.8** ± 2.2	1.1^#^ ± 0.2
Garr	Bare	5.1e+08 ± 7.6e+07	3.4e+13 ± 4.9e+12	1.7e+10^NS^ ± 2.0e+09	3.9e+09^NS^ ± 5.5e+08	2.0e+10 ± 3.0e+09	2.8e+11 ± 4.4e+10	1.4e+10 ± 2.1e+09	3.1e+11 ± 4.8e+10	59.7^#^ ± 3.8	3.7* ± 0.1	26.3 ± 1.8	2.6 ± 0.1	6.2 ± 1.2	0.3 ± 0.1
	Seagrass	1.2e+09** ± 3.7e+08	1.0e+14** ± 1.7e+13	2.2e+10^NS^ ± 8.5e+09	7.2e+09^NS^ ± 1.7e+09	4.2e+10^#^ ± 7.4e+09	6.4e+11* ± 1.1e+11	5.3e+10^#^ ± 1.6e+10	7.3e+11* ± 1.3e+11	47.3 ± 5.2	3.4 ± 0.0	30.8^#^ ± 2.7	2.8^#^ ± 0.1	10.0* ± 0.9	0.6* ± 0.1
Gail	Bare	1.8e+09^NS^ ± 4.1e+08	6.6e+13^NS^ ± 4.5e+12	1.8e+10* ± 6.9e+09	6.0e+09^NS^ ± 8.1e+08	3.1e+10^NS^ ± 3.7e+09	4.6e+11^NS^ ± 4.6e+10	2.4e+10^NS^ ± 5.1e+09	5.1e+11^NS^ ± 5.3e+10	35.8 ± 3.5	2.7 ± 0.1	26.5^NS^ ± 1.1	2.9^NS^ ± 0.1	4.2^NS^ ± 0.7	0.5^NS^ ± 0.1
	Seagrass	1.3e+09^NS^ ± 3.0e+08	5.8e+13^NS^ ± 7.8e+12	8.5e+09 ± 8.8e+08	4.8e+09^NS^ ± 4.7e+08	2.7e+10^NS^ ± 2.8e+09	3.6e+11^NS^ ± 3.5e+10	2.2e+10^NS^ ± 2.8e+09	4.1e+11^NS^ ± 4.0e+10	64.7* ± 14.3	3.4** ± 0.2	27.7^NS^ ± 2.9	2.9^NS^ ± 0.1	6.7^NS^ ± 1.4	0.6^NS^ ± 0.1
Ande	Bare	8.0e+08 ± 7.2e+07	2.1e+14^NS^ ± 2.5e+13	5.9e+10** ± 8.9e+09	2.5e+10^#^ ± 2.9e+09	9.5e+10^NS^ ± 8.5e+09	1.6e+12* ± 1.1e+11	1e+11^NS^ ± 1.3e+10	1.8e+12^#^ ± 1.3e+11	38.7** ± 2.9	2.9^NS^ ± 0.2	26.8 ± 0.8	2.9 ± 0.0	12.0^NS^ ± 2.1	0.7 ± 0.1
	Seagrass	1.2e+09^#^ ± 1.8e+08	1.7e+14^NS^ ± 3.5e+13	1.6e+10 ± 3.0e+09	1.6e+10 ± 2.8e+09	7.6e+10^NS^ ± 8.7e+09	1.2e+12 ± 1.2e+11	1e+11^NS^ ± 1.4e+10	1.4e+12 ± 1.4e+11	23.3 ± 1.9	2.7^NS^ ± 0.0	34.0* ± 2.0	3.1^#^ ± 0.1	17.0^NS^ ± 1.9	1.5** ± 0.1
All	Bare	8.5e+08 ± 1.0e+08	1.4e+14^NS^ ± 1.8e+13	2.2e+10^NS^ ± 3.3e+09	1.4e+10^NS^ ± 1.7e+09	6.6e+10^NS^ ± 7.5e+09	9.0e+11^NS^ ± 1.1e+11	5.9e+10 ± 7.7e+09	1.0e+12^NS^ ± 1.2e+11	38.3^NS^ ± 3.5	3.0^NS^ ± 0.1	26.9 ± 1.0	2.8 ± 0.0	7.4 ± 0.6	0.5 ± 0.0
	Seagrass	1.5e+09*** ± 1.6e+08	1.6e+14^NS^ ± 1.4e+13	2.1e+10^NS^ ± 2.5e+09	1.1e+10^NS^ ± 1.0e+09	6.5e+10^NS^ ± 5.9e+09	9.3e+11^NS^ ± 7.3e+10	7.1e+10^#^ ± 6.3e+09	1.1e+12^NS^ ± 8.4e+10	34.3^NS^ ± 3.2	2.9^NS^ ± 0.1	31.2*** ± 0.8	3.0*** ± 0.0	10.2** ± 0.8	0.8*** ± 0.1

*Note:* Statistical comparisons between bare and vegetated sediments were performed using Wilcoxon tests and significance levels are indicated as follows: ^#^ *p* < 0.1, *p < 0.05, **p < 0.01, ***p < 0.001, NS = not significant.

### 3.2 Key Parameters on CH_4_‐Cycling Microbial Communities

The abundance and diversity of the CH_4_‐cycling genes showed different patterns depending on sediment Type and site location. Sediments located in sheltered sites far from the ocean inlet hosted the more abundant CH_4_‐cycling prokaryotic communities with higher abundances of *pmoA*, *mmoX*, and *mcrA* (also for the overall bacterial communities), while vegetated sediments hosted more diverse methanotrophic communities (Figure 2, Table 2, Table S3a). In all studied sediments, *pmoA* was the more abundant functional gene (1.0^12^ ± 7.3^10^ copies per m^2^) followed by *mcrA* (2.1^10^ ± 2.1^09^ copies per m^2^) and by *mmoX* (1.2^10^ ± 1.0^9^ copies per m^2^) (*p* < 0.001). The diversity and richness followed a different order being higher in *mcrA* followed by *mmoX* and by *pmoA* (*p* < 2 × 10^−16^, Table 2, Table S3b, Figure S2).

**FIGURE 3:**
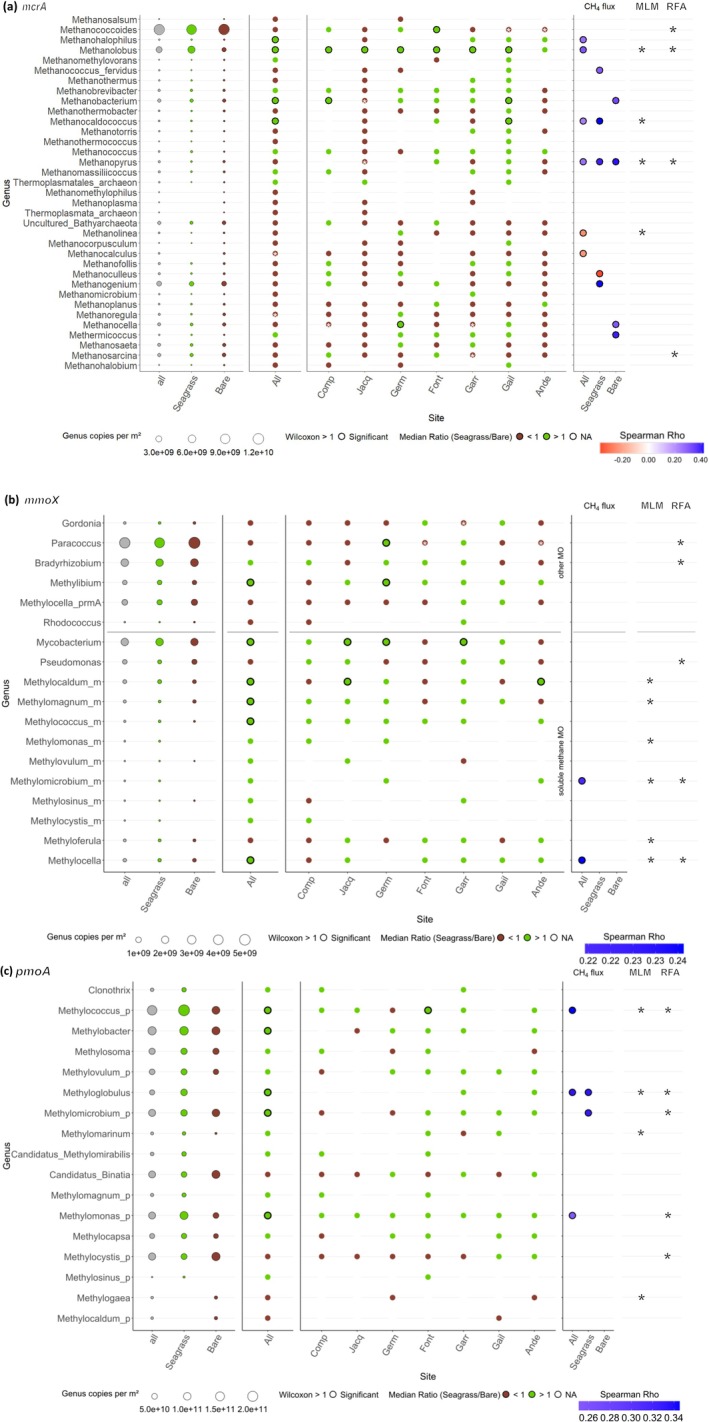
Differential abundance and relationships with CH_4_ flux for methanogenic and methanotrophic genera across seagrass and bare sediments in Arcachon Bay. Panel (a) includes genera identified from the *mcrA* gene (methanogenesis marker), panel (b) from *mmoX*, and panel (c) from *pmoA* (methanotrophy markers). For each genus, the first three columns show mean abundance (gene copies per m^2^, scaled by point size) across all samples, seagrass, and bare sediments, *n* = 83. The next two panels display, respectively, overall and site‐specific median abundance ratios (Seagrass/Bare), color‐coded (green: ratio > 1, seagrass affinity; brown: ratio < 1, bare affinity). Sites are ordered by increasing CH_4_ fluxes measured in seagrass sediments. Significant differences (Wilcoxon test, *p* < 0.05) are indicated by a black circle for seagrass preference and a white star within the brown point for bare preference. The penultimate panel shows only significant Spearman correlations (Rho) between genus abundance and CH_4_ flux, with colors indicating correlation strength and direction (blue: positive, red: negative). The final panel displays genera identified as important markers of CH_4_ flux based on mixed linear models (MLM) and random forest analysis (RFA), indicated by asterisks. “NA” denotes genera absent in both habitats.

Across the bay, vegetated sediments showed higher CH_4_ fluxes, higher total DNA content, and distinct CH_4_‐cycling microbial communities compared to bare sediments (Tables 1 and 2), although this pattern varied among sites. For *mcrA*, 7 of 36 genera showed significant associations, 5 enriched in seagrass (i.e., *Methanolobus*, *Methanobacterium*, *Methanohalophilus*, *Methanocaldococcus*), and 2 enriched in bare sediments (i.e., *Methanoregula*, *Methanocalculus*) (Figure 3a). For *mmoX*, 6 of 18 genera were enriched in seagrass (i.e., *Mycobacterium*, *Methylibium*, *Methylocaldum*, *Methylomagnum*, *Methylococcus*, *Methylocella*), while none were specific to bare sediments (Figure 3b). Among the 17 *pmoA* genera, five (i.e., *Methylococcus*, *Methylobacter*, *Methyloglobulus*, *Methylomicrobium*, *Methylomonas*) were both significantly associated with seagrass and abundant (Figure 3c).

**FIGURE 4:**
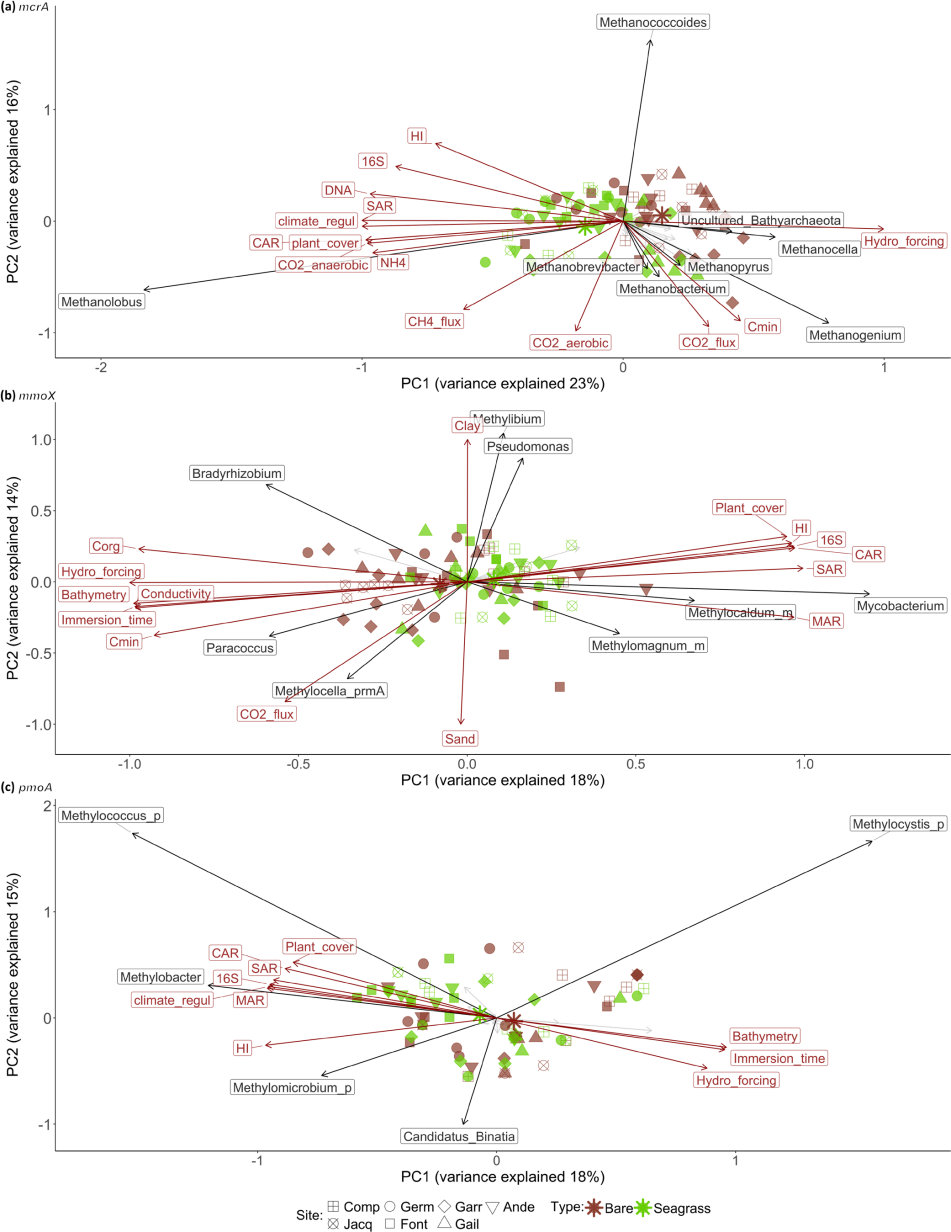
Principal component analysis (PCA) biplots illustrating the relationships between microbial genera (based on functional genes) and environmental variables across all sampling sites. The analyses are based on the absolute abundance of genera associated with the genes: (a) *mcrA*, (b) *mmoX*, and (c) *pmoA*. Only microbial genera with significant contributions to PC1 and PC2 are displayed in black, and only environmental variables with significant correlation with components are shown in red. Sites are represented by different shapes, and symbol colors denote sediment Type, *n* = 83. Larger star symbols indicate the barycenter of each Type.

Ordination of microbial communities based on absolute genus abundances (Figure 4) mirrored the global PCA combining environmental, microbial, and GHG variables (Figure 2), with vegetation presence and related environmental variables emerging as the main structuring factors. Only a subset of genera correlated significantly with CH_4_ fluxes (Figure 3), and even fewer were specifically linked to CH_4_ production or oxidation (Figure S3). Moreover, the genera correlated with each of these processes were generally not the same. Four genera (*mcrA–Methanolobus*, *mmoX–Methylocella*, *pmoA–Methylococcus* and *–Methyloglobulus*) consistently emerged as key taxa (Figures 3 and 4), being abundant, seagrass‐associated, correlated with CH_4_ fluxes, and supported by MLM and RFA (marked with asterisks in Figure 3). However, the whole community (or its diversity) rather than specific genera seems to be marking the CH_4_ flux (details in the next section).

### 3.3 Modeling of CH_4_ Fluxes

Across all tested models, CH_4_ flux variability was consistently explained by a limited set of key descriptors, regardless of the statistical structure or variable subset considered. Overall, vegetation presence, sediment C‐related parameters (CO_2_ fluxes and CAR), and microbial methane‐oxidation potential (notably *pmoA*‐related metrics) emerged as the most influential descriptors of CH_4_ fluxes (Table 3). Among the spatial variability, only the presence of seagrass (sediment Type) explains 60% of the CH_4_ fluxes (model 10). Adding the Site variability nested in the sediment Type (model 2) increased the explanatory power to 74%, while the Site variability alone did not describe fluxes at all (0%, model 23).

**TABLE 3:** Summary of the best significant mixed linear models explaining methane (CH_4_) fluxes across different environmental conditions. Models are ranked by corrected Akaike information criterion (AICc), from lowest (best fit) to highest, and are grouped according to fixed or random factors: Site, sediment Type (bare vs. seagrass), Subsite and Site nested within Type. Within each random factor structure of the combined dataset, the top‐performing models are shown in bold. All models include CH_4_ flux as the response variable, with physical, gas‐related, chemical, or microbial variables used as predictors. Only the best model(s) per subset are presented here and *n* = 83. Models were fitted using linear models (LM) or linear mixed‐effects models (LME), depending on the inclusion of random effects. For model selection purposes only, models without random effects (LM) were refitted using generalized least squares (GLS), allowing comparison with mixed‐effects models on a consistent likelihood basis. AICc values were computed from these GLS formulations for model comparison across all models. These models exhibited higher AICc values than the corresponding models with an empty fixed‐effect structure. In cases of similar AICc values, the simpler model was retained (see Table S2m for alternative models).

Model	Formula: Fixed part	Random part	AICc (GLS)	Fixed *R* ^2^	Global *R* ^2^	Subset
**1**	**log**_**10**_ **(CH**_**4**_ **_flux) = 0.30 × CO**_**2**_ **_flux**	**~1|Type/Site**	**183**	**0.06**	**0.76**	Combined
2		~1|Type/Site	187	0	0.74	Combined
**3**	**log**_**10**_ **(CH**_**4**_ **_flux) = 0.52 × CAR + 0.36 × CO**_**2**_ **_flux**	**~1|Subsite**	**187**	**0.34**	**0.69**	Combined
**4**	**log**_**10**_ **(CH**_**4**_ **_flux) = 0.33 × CAR + 0.30 × CO**_**2**_ **_flux − 0.24 × HI**	**~1|Type**	**189**	**0.17**	**0.63**	Combined
5	log_10_(CH_4__flux) = 0.35 × CO_2__flux	~1|Subsite	190	0.11	0.69	Gas
6	log_10_(CH_4__flux) = 0.48 × CAR	~1|Subsite	192	0.22	0.65	Chemical
7	log_10_(CH_4__flux) = 0.25 × sqrt(pmoA_R) − 0.24 × mmoX_D − 0.22 × log_10_(DNA)	~1|Type	193	0.09	0.76	Microbial
8		~1|Subsite	194	0	0.64	Subsite
9	log_10_(CH_4__flux) = 0.25 × CO_2__flux	~1|Type	196	0.05	0.64	Gas
10	log_10_(CH_4__flux) = 0.27 × CAR − 0.22 × HI	~1|Type	199	0.07	0.59	Chemical
11	log_10_(CH_4__flux) = 0.21 × Sand	~1|Type	200	0.03	0.67	Physical
12		~1|Type	201	0	0.60	Type
**13**	**log**_**10**_ **(CH**_**4**_ **_flux) = 0.82 × CAR + 0.58 × CO**_**2**_ **_flux**	**~1|Site**	**212**	**0.51**	**0.69**	Combined
**14**	**log**_**10**_ **(CH**_**4**_ **_flux) = 0.64 × CAR + 0.46 × CO**_**2**_ **_flux − 0.25 × HI − 0.24 × Sand + 0.22 × *Methylomicrobium_m***		**193 (212)**	**0.47**		Combined
15	log_10_(CH_4__flux) = − 1.74 × Hydro_forcing + 0.94 × Bathymetry − 0.78 × DBD	~1 + DBD|Site	202	0.50	0.88	Physical
16	log_10_(CH_4__flux) = 0.61 × CAR	~1|Site	225	0.32	0.40	Chemical
17	log_10_(CH_4__flux) = 0.54 × CAR − 0.22 × HI		216 (225)	0.26		Chemical
18	log_10_(CH_4__flux) = 0.41 × sqrt(pmoA_R)		226 (231)	0.16		Microbial
19	log_10_(CH_4__flux) = −0.57 × Hydro_forcing − 0.46 × DBD		224 (232)	0.18		Physical
20	log_10_(CH_4__flux) = 0.40 × log_10_(CO_2__anaerobic)		226 (232)	0.15		Gas
21	log_10_(CH_4__flux) = 0.43 × log_10_(CO_2__anaerobic)	~1|Site	233	0.18	0.22	Gas
22	log_10_(CH_4__flux) = 0.41 × sqrt(pmoA_R)	~1|Site	234	0.16	0.16	Microbial
23		~1|Site	243	0	0.00	Site

**FIGURE 5:**
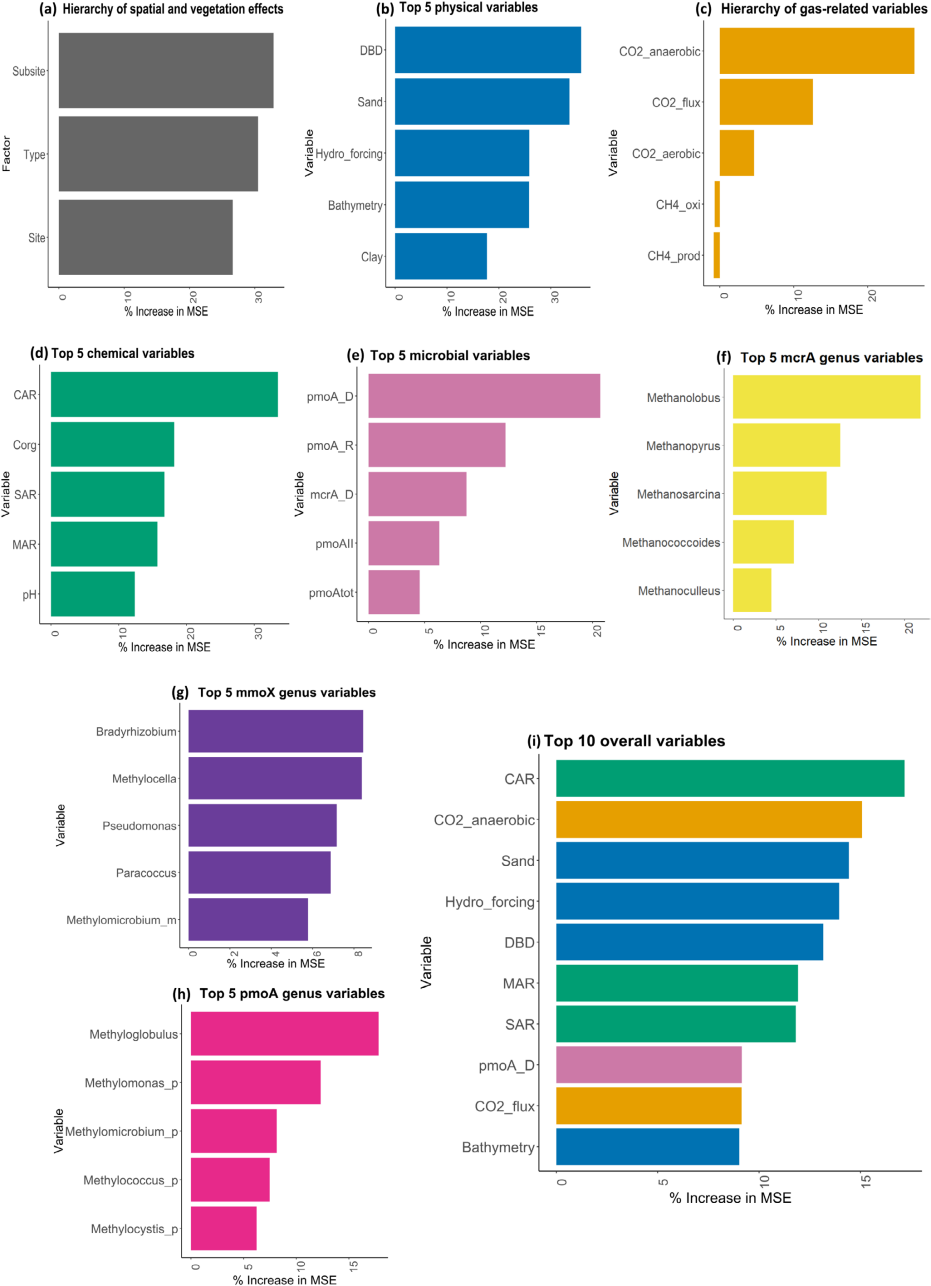
Variable importance in random forest analysis identifying key descriptors of methane (CH_4_) fluxes. (a) Hierarchy of spatial and vegetation effects (Site, sediment Type, and Subsite). (b–d) Top 5 predictors within each environmental category: (b) physical, (c) gas‐related, and (d) chemical variables, *n* = 83. (e) Top 5 microbial predictors based on functional gene abundances, diversity indices, 16S rRNA gene copies, and DNA content. (f–h) Top 5 taxonomic predictors at the genus level for *mcrA*, *mmoX*, and *pmoA* genes, respectively. (i) Overall top 10 predictors of CH_4_ flux across all variables.

C‐related and gas exchange descriptors were consistently among the strongest descriptors of CH_4_ fluxes. In particular, CO_2_ fluxes and CAR were repeatedly retained in the best‐performing models, showing positive relationships with CH_4_ emissions. These variables appeared in both simple and complex model structures, including gas‐only and chemical‐only models (models 5, 6, and 9), as well as in the best final models with a combined subset of descriptors (models 1, 3, 4, 13, and 14) explaining up to 76% of the variance. Lability of OM (HI) showed a negative link to the CH_4_ fluxes (models 4, 10, and 17). The RFA overall agreed with the models, confirming CAR and CO_2_ fluxes as high‐influencing variables, but also highlighting the importance of the positive relationship of anaerobic CO_2_ production (Figure 5i), which was included in some of the best models with gas variables (Table S2b) but not in the final models.

Physical sediment descriptors also contributed to the CH_4_ flux variability. Hydrodynamic forcing and sediment dry bulk density were negatively related to CH_4_ fluxes, whereas bathymetry showed a positive effect, indicating higher emissions in deeper environments (explaining up to 88% of the variance, model 15). In addition, sand content was positively associated with CH_4_ fluxes in vegetated sediments (model 11).

The best microbial model (model 7, explaining up to 76% of the variance) showed a positive association between CH_4_ fluxes and *pmoA* richness, while *mmoX* diversity and total DNA content were negatively related. The mentioned diversity metrics are more informative than functional gene abundance (qPCR) or genus‐level abundance, resulting in more explanatory models and RFA outputs, with the *pmoA* gene emerging as the most influential descriptor (Figure 5e–i, Table S2d–i, Figure S4a,b).

## 4 Discussion

### 4.1 Low CH_4_ Fluxes in Arcachon Bay Compared to Other Seagrass Meadows

We observed higher CH_4_ fluxes in seagrass‐vegetated areas compared to bare sediments, agreeing with previous studies (Al‐Haj and Fulweiler 2020; Tan et al. 2025). Two recent syntheses comparing CH_4_ fluxes across the sediment–water–air continuum in seagrass ecosystems reported median values of 64.8 and 35.8 μmol CH_4_ m^−2^ day^−1^, respectively (Al‐Haj and Fulweiler 2020; Eyre et al. 2023). In comparison, we observed an average flux of 24.4 ± 2.6 μmol CH_4_ m^−2^ day^−1^ in vegetated zones, with a median of 13.7 μmol CH_4_ m^−2^ day^−1^, is lower than both values but still indicates a significant contribution of seagrass sediments to atmospheric CH_4_ emissions. The measured CH_4_ fluxes represent 6.1% ± 0.8% of the estimated C burial in the Arcachon Bay (Dolivet‐Maréchal et al. 2026). These results highlight the need to consider both long‐term sequestration and emission processes when evaluating the climate regulation potential of blue C habitats. Importantly, comparisons with global syntheses should be interpreted with caution, as highlighted in recent critical discussions of methane meta‐analyses in coastal vegetated ecosystems (Rosentreter and Williamson 2020). These authors emphasize that reported flux distributions are often highly skewed and sensitive to methodological choices, spatial and temporal coverage, and the use of mean versus median statistics for upscaling. Such considerations may partly explain differences between our site‐specific estimates and global synthesis values. Our reported values should therefore be interpreted as snapshot measurements under standardized daytime low‐tide conditions rather than integrated daily fluxes.

Our measurements were performed at midday during low tide, using opaque chambers on exposed sediments to standardize sampling across sites. While this approach likely captured near‐maximum emission rates, it does not fully account for diel or tidal variability, which can influence CH_4_ release through changes in sediment oxygenation, water coverage, and temperature (Deborde et al. 2010; Maher et al. 2015; Rosentreter et al. 2021). In some temperate seagrass meadows, CH_4_ fluxes peak in the afternoon and evening rather than at night, suggesting that thermal and physical drivers can partially override redox‐dependent diel patterns (Henriksson et al. 2024). Thus, while our measurements provide robust estimates of relative differences between vegetated and bare sediments, continuous in situ monitoring would be valuable to capture short‐term dynamics and further refine flux estimates.

The observation of overall negative CO_2_ fluxes likely reflects a net ecosystem CO_2_ uptake during the measurement period. Although opaque chambers were used to minimize direct light effects, measurements were conducted at midday during low tide when benthic primary producers, including seagrass and microphytobenthos, may still sustain high photosynthetic activity prior to full chamber equilibration. This can result in a net drawdown of dissolved CO_2_ within the sediment–water–air system, leading to negative flux estimates even under partially light‐limited conditions. Net CO_2_ uptake was observed at the study site during daytime low‐tide conditions measuring with Eddy covariance tower (Polsenaere et al. 2012). In addition, CO_2_ dynamics in such environments are strongly influenced by carbonate system equilibria and gas exchange between porewaters and the overlying atmosphere, which can further contribute to net CO_2_ uptake signals during low‐tide conditions. These CO_2_ fluxes therefore reflect short‐term net ecosystem metabolism under daytime low‐tide conditions, however using a transparent chamber will be more proper to measure CO_2_ flux in light conditions.

Seagrass meadows in our study area have declined by approximately 50% since the 1990s (Muller et al. 2024). As restoration efforts to increase seagrass distribution may enhance CH_4_ emissions alongside C burial, future studies should explore how meadow age, species, coverage, and biomass per area may influence flux variability. These factors can influence CH_4_ production and oxidation through changes in organic matter availability, plant‐mediated oxygen transport, and sediment redox conditions, while also affecting carbon burial through variations in primary production and sediment accumulation (Asplund et al. 2022; Yau et al. 2023; Henriksson et al. 2024). Moreover, a dedicated study on N_2_O emissions, using a similar approach to ours, would also be valuable, given its high warming potential (273 times greater than CO_2_) (IPCC 2023) and the current lack of data (Murray et al. 2015).

### 4.2 C‐Related Features as Key Descriptors of CH_4_ Fluxes

The combined use of MLM and RFA identified C‐related variables as the most consistent and important descriptors of CH_4_ fluxes across our sites, supporting our initial hypothesis that CH_4_ fluxes are influenced by sedimentary OM, particularly the C related traits. CO_2_ flux and CAR positively influenced CH_4_ emissions, while lability of OM (HI) showed negative associations. Importantly, the positive effect of CO_2_ flux in the models reflects the interpretation of flux magnitude within a standardized analytical framework, where higher CO_2_ flux values correspond to reduced net CO_2_ uptake (i.e., less negative fluxes). This indicates that sites with lower CO_2_ uptake and higher carbon accumulation tend to exhibit higher CH_4_ emissions.

Our results suggest that C‐rich environments with more recalcitrant OM tend to support higher CH_4_ emissions. This is possibly due to more C in deeper sediments, where anaerobic conditions are conducive to methanogenesis and sustained microbial activity. The negative relationship with lability (HI) supports the idea that labile OM is preferentially degraded through aerobic or alternative anaerobic pathways, reducing CH_4_ production (Le Mer and Roger 2001).

Also, the particle size matters, as finer particles and more C_org_ promote both C storage and CH_4_ emissions (Al‐Haj and Fulweiler 2020; Eyre et al. 2023). This can be explained, in part, by a positive feedback loop in seagrass ecosystems: the presence of vegetation slows down water flow, enhancing the deposition of fine particles and organic matter. While this effect is spatially variable across sites (with only 3 out of 7 sites showing higher clay content in vegetated sediments), a bay‐scale comparison between seagrass and bare sediments still revealed an overall enrichment in fine particles in vegetated areas. This further stabilizes the sediments (Cognat 2019) and fosters anaerobic conditions favorable to methanogenesis despite the O_2_‐extrusion in the roots. Indeed, our best‐performing model with site as a random factor highlighted the negative role of hydrodynamic forcing in modulating CH_4_ fluxes, agreeing with Jin et al. (2025). Stronger hydrodynamics increases sediment mixing, which disrupts anaerobic microsites and favors CH_4_ oxidation or alternative heterotrophic metabolism, and could even increase sediment instability till avoiding seagrass presence and C burial.

Importantly, the presence of seagrass alone explained a large proportion (60%) of the variance in CH_4_ fluxes. This suggests that vegetation not only facilitates C accumulation but also drives biogeochemical processes through its influence on sediment characteristics and microbial communities.

Overall, our results indicate that while increased C burial represents a clear benefit for climate regulation, it is accompanied by a disservice in the form of higher CH_4_ emissions. Nevertheless, these processes appear positively associated, as sites with greater C burial also tend to exhibit higher CH_4_ emissions. Overall, our results suggest that C burial and CH_4_ emissions are not in a simple trade‐off relationship, but rather tend to co‐occur across sites. All C‐related variables, except HI, were positively associated with CH_4_ fluxes, indicating a cumulative effect of C quantity on both sequestration and emission. Therefore, promoting C storage in these systems may inherently involve accepting increased CH_4_ emissions, a conclusion supported by both our statistical models and the PCA. This dual role of vegetation, enhancing both C sequestration and CH_4_ production, is consistent with earlier observations (Macreadie et al. 2019; Eyre et al. 2023; Kristensen et al. 2025; Dolivet‐Maréchal et al. 2026) and reinforces the complexity of blue C system dynamics.

### 4.3 Diversity of Methanotrophs Is the Best Microbial CH_4_ Flux Descriptor

Modeling revealed that functional diversity of CH_4_‐cycling genes was a better descriptor of CH_4_ fluxes than functional gene abundance or than the presence of specific genus of microbes carrying CH_4_‐cycling genes. Similarly, methanotroph diversity was a better molecular descriptor than gene abundance (qPCR) when modeling CH_4_ fluxes in rivers (Saarela et al. 2026). The most explanatory microbial model identified *pmoA* richness as a positive descriptor, while *mmoX* diversity and total DNA content were negatively associated with CH_4_ emissions. Also, we observed significant and positive correlations between each of the *pmoA* and *mmoX* genera and CH_4_ fluxes, these genera being more abundant in vegetated than bare sediments. These findings suggest that diverse methanotrophic communities in CH_4_‐rich environments may help buffer CH_4_ release by enhancing oxidation capacity (Arnold et al. 2023; Knief 2015; Tan et al. 2025).

Our results show that microbial communities differ between bare and seagrass sediments, with the key genera highlighted in this study being more abundant in vegetated sites. In addition, methanotroph diversity is higher in vegetated sediments. However, contrary to our initial hypothesis, the abundance and diversity indices of methanogens were not significantly higher in vegetated sediments compared to bare sediments. Furthermore, *mcrA* variables were not included in models of CH_4_ fluxes, agreeing with other studies with no clear links between methanogens and CH_4_ production potential (Berberich et al. 2020; Chaudhary et al. 2017). This may reflect the fact that methanogenesis can significantly occur also at deeper anoxic layers, which were not included in our 0–10 cm sediment sampling. A more stratified and deeper sampling approach will be needed to help distinguish vertical patterns in community structure and activity. In contrast, we observed higher diversity and richness of *pmoA‐* and *mmoX‐*methanotrophs in seagrass sediments compared to unvegetated ones, a pattern also reported by Tan et al. (2025). Vegetation likely promotes the development of diverse methanotrophic communities by enhancing sediment oxygenation via root O_2_ release (Brodersen et al. 2024) and photosynthetic O_2_ production, both of which can stimulate CH_4_ oxidation (Lyimo et al. 2018).

Although taxonomic identity alone explained less variation in CH_4_ fluxes than functional diversity, certain genera stood out. *Methanolobus* (*mcrA*) was particularly notable, being also more abundant in vegetated than in bare sediments in the Chinese coast (Tan et al. 2025) and contributing to CH_4_ production in brackish Baltic Sea sediments (Tsola et al. 2024), consistent with our observations showing a positive correlation with in situ CH_4_ fluxes. Aerobic methanotrophic genera such as *Methylococcus* and *Methylomicrobium*, which harbor both *mmoX* and *pmoA* genes and belong to the *Gammaproteobacteria* class, have also been reported to be strongly associated with seagrass sediments (Tan et al. 2025). *Methylococcus* has been identified as a predominant aerobic methanotroph in marine sediments (Håvelsrud et al. 2011), in line with our results showing it as the most abundant genus for the *pmoA* gene. In addition, *Methylocella*, a facultative methanotroph capable of using CH_4_ as well as multicarbon substrates such as acetate, pyruvate, and malate, has been observed across a wide range of ecosystems, including lake and marine sediments (Rahman et al. 2011), and was also detected in our marine sediments, showing a preference for vegetated areas and a positive correlation with in situ CH_4_ fluxes. Similarly, *Methyloglobulus* has been associated with aquatic rhizospheres and identified as one of the most abundant methanotrophs carrying the *pmoA* gene, consistent with our observations in vegetated sediments, suggesting a potential role in CH_4_ oxidation within root‐associated aquatic zones (Bao et al. 2025). These findings reinforce the idea that seagrass habitats support complex and spatially coupled CH_4_ production and oxidation processes.

Our approach enabled a comprehensive characterization of key CH_4_‐cycling microbial groups, but some technical limitations remain. For qPCR, the *pmoA* gene was targeted with three primer sets, whereas *mmoX* and *mcrA* were each amplified with a single primer set, which may have contributed to the higher *pmoA* abundance observed. Similarly, reference databases are more complete for *mcrA*‐associated methanogens than for methanotrophs, potentially affecting richness and diversity estimates, although *mmoX* values were also high despite the smaller database. These limitations highlight the importance of balanced primer design and curated databases (Campbell et al. 2022). Future studies integrating RNA‐ or protein‐based analyses could identify metabolically active microbes and provide functional insights into in situ CH_4_‐cycling (Markovski et al. 2025).

## 5 Conclusion

This study provides new insights into the biogeochemical functioning of seagrass ecosystems by showing that CH_4_ emissions, although generally modest, are highly influenced by C levels. Seagrass presence enhances CH_4_ fluxes through organic C accumulation, finer sediment particles and reduced hydrodynamic forcing. Vegetated sediments also showed more diverse methanotrophic communities. These CH_4_ emissions can co‐occur with CO_2_ fluxes and high blue C sequestration, reflecting a complex ecological trade‐off rather than a simple dichotomy. As such, seagrass restoration and management strategies should consider both components of their climate impact, namely carbon burial and associated CH_4_ emissions, as these processes co‐occur and jointly determine the net climate effect of these ecosystems. To better quantify this balance, future research should integrate C burial with high‐resolution flux monitoring and activity‐based microbial profiling, in order to improve the prediction of site‐specific net greenhouse gas budgets.

## Author Contributions

**Marion Dolivet‐Maréchal:** conceptualization, investigation, writing – original draft, methodology, visualization, writing – review and editing, software, formal analysis, data curation, resources, project administration, validation, supervision. **Carlos Palacin‐Lizarbe:** writing – review and editing, supervision, methodology, software, data curation, validation, formal analysis, visualization, resources. **Henri M. P. Siljanen:** resources, writing – review and editing, software, methodology, validation, supervision, data curation. **Dhiraj Paul:** methodology, software, data curation, resources. **Abigaïl Delort:** methodology, validation, writing – review and editing, resources, investigation. **Jonathan Gervaix:** resources, methodology, validation, writing – review and editing, investigation. **Charline Creuzé des Châtelliers:** investigation, writing – review and editing, resources, methodology, validation. **Sabine Schmidt:** investigation, writing – review and editing, methodology, validation, data curation, supervision, resources, formal analysis, visualization. **Mathis Cognat:** investigation, methodology, validation, visualization, resources. **David Sebag:** investigation, validation, methodology, writing – review and editing, data curation, resources, formal analysis. **Olivier Taugourdeau:** resources, writing – review and editing. **Clara Schübert:** investigation, resources. **Nathalie Labourdette:** investigation, resources, data curation, validation, writing – review and editing. **Isabelle Bertrand:** resources, data curation. **Lorenzo M. W. Rossi:** resources, data curation. **Xavier Le Roux:** conceptualization, investigation, funding acquisition, methodology, project administration, resources, supervision, validation. **Agnès Richaume:** writing – review and editing, validation, resources, supervision, methodology, project administration. **Alessandro Florio:** investigation, writing – review and editing, validation, methodology, project administration, supervision, resources.

## Funding

This work was supported by the Green Deal project REST‐COAST, funded by the European Union's Horizon 2020 research and innovation programme (Grant Agreement 101037097). Additional support was provided by the Graduate School H2O'Lyon (ANR‐17‐EURE‐0018) of Université de Lyon (UdL), within the program “Investissements d'Avenir” operated by the French National Research Agency (ANR).

## Conflicts of Interest

The authors declare no conflicts of interest.

## Supporting information


**Figure S1:** Principal component analysis (PCA) biplot illustrating the relationships among physical, chemical, microbial, and GHG variables across seven sites (*n* = 83 samples), accompanied by a map of Arcachon Bay. Arrows indicate the contribution of each variable to the ordination. Both axes were rescaled (×6) to improve visualization of sites and variables. Sites are represented by different shapes and colors, with larger symbols denoting the barycenter of each site. The map uses the same color scheme for each site as the PCA plot, facilitating comparison. This figure confirms the ordination pattern shown in Figure 2 of the main text. Together, the PCA and map highlight the PC1 gradient, which reflects a large‐scale ecological gradient linked to ocean connectivity and sediment stability. Sites closer to the ocean inlet, located on the left side of both figures, are characterized by longer immersion times, stronger current flows, and greater depths (higher bathymetry), such as Gail and Garr. In contrast, the sites Ande and Font, positioned on the right side of PC1, stand out as distinct and more isolated. These sites lie in the inner part of the bay, indicating a lower degree of connection to the open ocean.
**Figure S2:** Comparison of CH_4_ fluxes, CH_4_‐related processes, and microbial gene features across sites and sediment types in Arcachon Bay. Panels (a–c) show measured CH_4_ flux (a), potential CH_4_ production (b), and CH_4_ oxidation rates (c) across sites. Panels (d–f) display mcrA gene copy numbers (d), richness (e), and diversity (f). Panels (g–i) show equivalent metrics for mmoX gene (g) copy number, (h) richness, (i) diversity. Panels (j–l) show pmoA gene copy numbers (j), richness (k), and diversity (l). Each bar represents mean ± standard error (*n* = 6 per subsite; *n* = 5 for Comp‐Bare). Colors indicate sediment type: green = seagrass, brown = bare. Significant differences (Wilcoxon tests) between bare and vegetated sediments are indicated as follows: ^#^ *p* < 0.1, *p < 0.05, **p < 0.01.
**Figure S3:** Significant Spearman correlations (Rho) between genus abundances and CH_4_‐related gases. Panel (a) shows genera identified from the mcrA gene (a methanogenesis marker), panel (b) from mmoX, and panel (c) from pmoA (both methanotrophy markers). For each gene, the first column lists genera significantly correlated with CH_4_ flux, the second column with CH_4_ production, and the third with CH_4_ oxidation. Colors indicate the strength and direction of the correlation (blue = positive, red = negative), *n* = 83.
**Figure S4:** Variable importance in random forest analysis identifying key microbial predictors of methane (CH_4_) fluxes. Importance was assessed using the mean increase in mean squared error (% Increase in MSE) after permutation of each variable, *n* = 83. (a, b) Top 5 predictors within each microbial category: (a) qPCR‐based variables, (b) diversity indices. (c–e) Hierarchical importance of microbial predictors associated with the mcrA, mmoX, and pmoA genes, respectively.
**Table S1:** Target genes, primer names and sequences, amplicon sizes, and references used for quantitative PCR. Total pmoA (pmoAtot) abundance was calculated by summing the gene copy numbers of the three pmoA clades, providing a comprehensive estimate of particulate methane monooxygenase gene abundance in the samples.
**Table S2:** Summary of significant mixed linear models explaining methane (CH_4_) fluxes across different variable subsets. Models within each subset are ranked and grouped according to fixed or random effects: site, sediment type (bare vs. seagrass), and subsite. The top‐performing models within each group (i.e., those with the lowest AICc) are highlighted in bold. All models use CH_4_ flux as the response variable, with predictors drawn from physical (a), gas‐related (b), chemical (c), microbial (d–l), or combined (m) variable categories. Only models containing significant (*p* < 0.05) or marginally significant (^#^ *p* < 0.1) predictors within each subset are presented. Variables included in each subset are listed at the top of their respective tables, *n* = 83. Models were fitted using linear models (LM) or linear mixed‐effects models (LME), depending on the inclusion of random effects. For model selection purposes only, models without random effects (LMs) were refitted using generalized least squares (GLS), allowing them to be compared on a consistent likelihood basis with mixed‐effects models. AICc values were then computed from these GLS formulations for model comparison across all models.
**Table S3:** Microbial characteristics of the studied sites. (a) DNA content and qPCR results. (b) Diversity indices. Values are expressed as mean ± standard error (SE), *n* = 83. Sites are ordered along a gradient from the most negative to the most positive values along PC1 (see Figure 2), which reflects a multivariate ordination of environmental and microbial descriptors. Differences among sites were assessed using nonparametric Kruskal–Wallis tests, followed by Dunn's post hoc tests with Benjamini–Hochberg correction for multiple comparisons. Different letters indicate significant differences among sites for each variable (*p* < 0.05); groups sharing at least one letter are not significantly different.
**Table S4:** Characteristics of the studied sites under bare and vegetated (seagrass) conditions, with variables grouped into general and physical features (a, b) and chemical variables (c, d). Values are reported as mean ± standard error (SE). The dataset includes a total of 83 observations, comprising 41 bare and 42 vegetated (seagrass) samples. Within each site, subsites represent spatially distinct sampling locations, each based on six replicate measurements, providing a hierarchical structure with measurements nested within subsites and subsites nested within sites. Significant differences (Wilcoxon tests) between bare and vegetated sediments are indicated as follows: ^#^ *p* < 0.1, *p < 0.05, **p < 0.01, ***p < 0.001, and NS (not significant), *n* = 83.

## Data Availability

The data used to calculate the MAR are archived on the SEANOE platform (https://doi.org/10.17882/99990). The Excel file and R code, which include the other variables used in this study and those for figure generation, are archived on Zenodo at https://doi.org/10.5281/zenodo.17243268. The Excel file contains data collected from seven sites within Arcachon Bay, and the R code includes statistical analyses (e.g., Wilcoxon tests, mixed linear models, and random forest analyses) as well as scripts for figure generation. Additionally, raw targeted metagenomic sequences generated in this study have been deposited in the NCBI Sequence Read Archive (SRA) under BioProject accession number PRJNA1335804.
