# Supplementary material for: Vegetation Increases CH 4 Emissions and Methanotroph Diversity in Marine Sediments
Source: Glob Chang Biol. 2026 Jul 7;32(7):e70989. doi: 10.1111/gcb.70989 (PMC13342776; doi:10.1111/gcb.70989)
Supplement: Supplementary file 1 — Figure S1: Principal component analysis (PCA) biplot illustrating the relationships among physical, chemical, microbial, and GHG variables across seven sites (n = 83 samples), accompanied by a map of Arcachon Bay. Arrows indicate the contribution of each variable to the ordination. Both axes were rescaled (×6) to improve visualization of sites and variables. Sites are represented by different shapes and colors, with larger symbols denoting the barycenter of each site. The map uses the same color scheme for each site as the PCA plot, facilitating comparison. This figure confirms the ordination pattern shown in Figure 2 of the main text. Together, the PCA and map highlight the PC1 gradient, which reflects a large‐scale ecological gradient linked to ocean connectivity and sediment stability. Sites closer to the ocean inlet, located on the left side of both figures, are characterized by longer immersion times, stronger current flows, and greater depths (higher bathymetry), such as Gail and Garr. In contrast, the sites Ande and Font, positioned on the right side of PC1, stand out as distinct and more isolated. These sites lie in the inner part of the bay, indicating a lower degree of connection to the open ocean. Figure S2: Comparison of CH4 fluxes, CH4‐related processes, and microbial gene features across sites and sediment types in Arcachon Bay. Panels (a–c) show measured CH4 flux (a), potential CH4 production (b), and CH4 oxidation rates (c) across sites. Panels (d–f) display mcrA gene copy numbers (d), richness (e), and diversity (f). Panels (g–i) show equivalent metrics for mmoX gene (g) copy number, (h) richness, (i) diversity. Panels (j–l) show pmoA gene copy numbers (j), richness (k), and diversity (l). Each bar represents mean ± standard error (n = 6 per subsite; n = 5 for Comp‐Bare). Colors indicate sediment type: green = seagrass, brown = bare. Significant differences (Wilcoxon tests) between bare and vegetated sediments are indicated as follows: # p < 0.1, *p [file GCB-32-e70989-s001.pdf]

Supplementary text

Materials and Methods

Characterisation of the sediments and sites

Plant cover

Seagrass (*Zostera noltii*) coverage was quantified following the European Water Framework Directive protocol (Auby et al., 2018). At each study point, a 30 × 30 cm quadrat was placed, photographed, and the percentage of seabed covered by seagrass was independently estimated by three researchers. The final coverage value for each quadrat was calculated as the average of these three estimates.

Hydrodynamic forcing

Hydrodynamic forcing was assessed using a dimensionless parameter, which quantifies the total hydrodynamic energy during a tidal cycle (spring or neap tide) that exceeds a critical velocity threshold for sediment erosion or leaf uprooting. This parameter was calculated following sediment transport model formulations (Le Hir et al., 2011), and applies to both vegetated and unvegetated areas. Further methodological details are available in Dolivet-Maréchal et al., 2026.

Analyses on the top 10 cm of mixed sediment cores

Abundance of bacterial 16S rRNA and functional CH_4-cycling genes

DNA was extracted from sediment samples using the NucleoSpin® Soil Kit (Macherey-Nagel GmbH & Co, Düren, Germany) (Béraud et al., 2025) following the manufacturer's protocol. DNA concentrations were quantified using a Qubit® 2.0 fluorometer and the Quant-iT™ dsDNA Broad Range (BR) Assay Kit (Invitrogen, France). The abundance of the bacterial community was estimated by absolute quantification of 16S rRNA gene copy number via quantitative real-time PCR (qPCR), using iTaq™ Universal SYBR® Green Supermix (Bio-Rad) on a CFX Connect

Real-Time PCR Detection System (Bio-Rad). The abundance of key genes involved in CH_4 production, i.e. *mcrA* (methanogenic archaea), and CH_4 consumption, i.e. *mmoX* (soluble methane monooxygenase), and three distinct clades of *pmoA* (particulate methane monooxygenase: *pmoAIa*, *pmoAIb*, and *pmoAII*) were also quantified. Detailed information on thermal conditions and primer sets is provided in Table S1. Melting curve analysis was conducted to verify PCR product specificity after amplification. Amplification showed a linear response ($R^2 > 0.99$) with no detectable PCR inhibitors, which were systematically checked.

Moisture

Moisture content of sediment samples was determined following ISO 11465 (gravimetric method: drying at 105 °C for 24 h and mass difference). Although this standard originates from soil sciences, it has been applied successfully in marine contexts (Shakeel et al., 2022), ensuring comparability and robustness of measurement.

pH

Sediment pH was measured using a protocol adapted from ISO 10390:2005. A 4 g portion of lyophilized sediment was placed into a 50 mL Falcon® tube, and 20 mL of deionized water were added. The suspension was mechanically shaken for 1 hour at 140 rpm and 20 °C. Samples were then left to rest at room temperature for 1 to 3 hours before measurement. After manual homogenization and pH meter calibration, the pH value was recorded either after 2 minutes or once the reading had stabilized.

Conductivity

Sediment conductivity was assessed following a procedure inspired by ISO 11265:1994. A 20 g aliquot of lyophilized sediment was placed into a 200 mL plastic flask, to which 100 mL of deionized water were added. The suspension was then vortexed vigorously five times at 30-minute intervals over a 2.5-hour period. After agitation, the mixture was filtered using a

Whatman No. 5 filter, and conductivity was measured on the resulting filtrate using a calibrated conductivity meter.

Sediment grain size

The sediment grain size distribution was measured using a Malvern laser diffraction instrument (LS13320XR), covering particle sizes from 0.4 to 2000 µm. Sediment samples (a few milligrams) were pretreated by removing organic matter with hydrogen peroxide and dispersed using sodium hexametaphosphate before analysis. The instrument's high sensitivity allowed small sample volumes to be used, ensuring accurate and efficient granulometry. The distribution of particle sizes was expressed as the percentage of clay (<2 µm), silt (2-50 µm), and sand (50-2000 µm).

Elementary analyser, nitrogen content

Total nitrogen (N) content was quantified using elemental analysis (e.g. Carlo Erba NA-1500 CNS analyzer) on decarbonated, dried, unsalted and homogenized sediment samples. The analyzer combusts the material at high temperature (1200°C), allowing precise measurement of nitrogen as a percentage of dry weight.

Nitrogen analyses

For organic nitrogen pool analysis, fresh soil samples (5 g dry weight equivalent) were transferred into 150 mL plasma vials. After determining the soil moisture content, a 0.01 M $CaCl_2$ solution (Houba et al., 2000) was added. The vials were sealed with parafilm (M®), and the soil suspensions were incubated at 20 °C with shaking (140 rpm) for 2 hours at 10 °C. Following incubation, the suspensions were filtered using membranes with a 0.22 µm pore size. Concentrations of NO_3^-, NO_2^-, and NH_4^+ were determined using a Smartchem 200 sequential analyzer (AMS Alliance, Villeneuve-la-Garenne, France).

Rock-Eval® Thermal analyses, hydrogen index

Rock-Eval® thermal analysis is a geochemical technique widely used to quantify and characterize the carbon content of sediment samples (Baudin et al., 2015). In this method, a small, dried, unsalted, and homogenized sediment sample undergoes two successive ramped heating steps: the first under an inert atmosphere (pyrolysis), followed by a second under an oxidative atmosphere.

During the pyrolysis phase, the sample is gradually heated from 200°C to 650°C. The thermal cracking of organic compounds releases a series of volatile products, mainly hydrocarbon effluents, carbon monoxide (CO), and carbon dioxide (CO_2), which are continuously monitored by flame ionization detector (FID) and infrared (IR) sensors. In the subsequent oxidation phase, the residual sample is heated from 200°C to 850°C in the presence of oxygen. This step enables the combustion of the remaining organic matter and the thermal decomposition of the mineral fraction, again releasing CO and CO_2, which are measured using IR detectors.

Based on the type, quantity, and temperature of gas release, Rock-Eval® allows the estimation of both total organic carbon (TOC [% g C·g^{-1}]) and mineral carbon (MinC [% g C·g^{-1}]). However, TOC values may sometimes be biased, particularly in non-carbonaceous samples, due to the misattribution of certain carbon signals, leading to an overestimation of the mineral carbon content. To correct for this analytical artifact, the SOTHIS correction method (Hazera et al., 2023; Sebag et al., 2022) was applied to the entire dataset. In the remainder of this article, *Cmin* refer to the MinC values corrected using the SOTHIS method.

In addition to quantitative carbon measurements, Rock-Eval® also provides qualitative indicators such as the Hydrogen Index (HI [mg HC·g^{-1} TOC]). HI is calculated as the ratio between the quantity of hydrocarbons released during pyrolysis (S2 peak) and the TOC content (HI = S2/TOC × 100, in mg HC·g^{-1} TOC). Higher HI values generally indicate hydrogen-

rich, well-preserved (typically autochthonous) organic matter, while lower values reflect more degraded or terrestrially derived inputs.

Analyses on the top 20 cm of undisturbed sediment cores

Sediment cores (up to 20 cm depth) were manually collected using 35 cm long PVC tubes inserted until resistance. In the lab, the cores were sectioned at 1 cm intervals, dried at 62 °C; wet and dry weights were used to calculate the dry bulk density (DBD), assuming a mineral density of 2.65 g cm^{-3}. The Sediment Accumulation Rate (SAR) (cm yr^{-1}) and Mass Accumulation Rate (MAR) (g cm^{-2} yr^{-1}) were calculated using excess ^{210}Pb (Pb_{xs}) profiles in the sediment, following Amann et al., 2023. A Broad-Energy (@Mirion) gamma spectrometer was used to measure ^{210}Pb, ^{226}Ra, and ^{232}Th at the EPOC laboratory. The excess of 210Pb ($210Pb_{xs}$), incorporated in sediments from atmospheric inputs and whose decrease with depth provides access to time, was obtained by subtracting ^{226}Ra from total ^{210}Pb and normalized using ^{232}Th. Sediment ages were estimated using the Constant Initial Concentration model(Robbins & Edgington, 1975). The SAR and MAR correspond to the slope of the depth-age and cumulative mass-age relationships, respectively.

C_{org} was measured in composite samples prepared from sediment layers located below the surface horizon affected by early diagenesis. Surface sediments were excluded to avoid bias due to organic matter degradation occurring within the uppermost centimeters. Following Delgard et al., 2013, who showed in Arcachon Bay that beyond 3-5 cm depth %C remains relatively constant down to 20 cm, we considered this deeper section as the “stable interval.” This choice is further supported by our sediment profiles in (Fig S2, Dolivet-Maréchal et al., 2026), which indicate a general stabilization of C_{org} values below the surface diagenetic layer (~3–5 cm) across most cores. Importantly, although excess ^{210}Pb profiles reveal substantial spatial variability in sediment mixing and accumulation—particularly in seagrass

sediments—this variability mainly affects the upper layers and does not contradict the observed stabilization of C_{org} at depth. Here, ^{210}Pb profiles were primarily used to reconstruct sediment chronology (SAR, MAR) rather than to define the C_{org} integration depth. Composite samples were prepared by pooling the selected layers within this interval for each core, with each layer weighted by its dry bulk density (DBD) to account for differences in sediment mass per unit volume. Sediments were ground, sieved (2 mm), and decarbonated following Lorrain et al., 2003. C_{org} content was determined using a Thermo FlashSmart Elemental Analyzer at the EPOC laboratory. CAR (g C m^{-2} yr^{-1}) was calculated as the product of MAR and %C_{org}, and converted to CO_2 equivalents using a 44:12 molar ratio. The net climate benefit of each habitat was assessed by balancing CAR against GHG emissions. Further details on this method are available in (Dolivet-Maréchal et al., 2026). These values (SAR, MAR and CAR) were calculated for the sediment layer corresponding to the last 17-year, the recent period of sedimentary stabilization

Supplementary reference

Amann, B., Chaumillon, E., Schmidt, S., Olivier, L., Jupin, J., Perello, M. C., & Walsh, J. P. (2023). Multi-annual and multi-decadal evolution of sediment accretion in a saltmarsh of the French Atlantic coast: Implications for carbon sequestration. *Estuarine, Coastal and Shelf Science*, *293*. https://doi.org/10.1016/j.ecss.2023.108467

Auby, I., Oger-Jeanneret, H., Gouillieux, B., Grall, J., Janson, A.-L., Maguer, M., Rigouin, L., Rollet, C., Sauriau, P.-G., & Trut, G. (2018). *Protocoles de suivi stationnel des herbiers à zostères pour la Directive Cadre sur l'Eau (DCE) Zostera marina-Zostera noltei*.

Baudin, F., Disnar, J. R., Aboussou, A., & Savignac, F. (2015). Guidelines for Rock-Eval analysis of recent marine sediments. *Organic Geochemistry*, *86*, 71–80. https://doi.org/10.1016/j.orggeochem.2015.06.009

Béraud, C., Piola, F., Gervaix, J., Boisselet, C., Creuze des Chatelliers, C., Defour, P., Delort, A., Derollez, E., Dumortier, L., Florio, A., Rasse, L., Vallier, F., & Cantarel, A. A. M. (2025). Unravelling knotweed clonal control of soil microbial activities related to the nitrogen cycle through plant growth phases and ramet positions within the patch. *Plant and Soil*. https://doi.org/10.1007/s11104-024-07190-9

Delgard, M. L., Deflandre, B., Deborde, J., Richard, M., Charbonnier, C., & Anschutz, P. (2013). Changes in Nutrient Biogeochemistry in Response to the Regression of Zostera noltii Meadows in the Arcachon Bay (France). *Aquatic Geochemistry*, *19*(3), 241–259. https://doi.org/10.1007/s10498-013-9192-9

Dolivet-Maréchal, M., Schmidt, S., Cognat, M., Schübert, C., Gervaix, J., Creuze des Chatelliers, C., Labourdette, N., Bertrand, I., Rossi, L. M., Dugue, R., Ahr, M., Richaume, A., Le Roux, X., & Florio, A. (2026). The Dual Climate Role of Seagrass Meadows in Arcachon Bay. *Earth Systems and Environment*. https://doi.org/10.1007/s41748-026-01029-2

Hazera, J., Sebag, D., Kowalewski, I., Verrecchia, E., Ravelojaona, H., & Chevallier, T. (2023). Adjustments to the Rock-Eval® thermal analysis for soil organic and inorganic carbon quantification. *Biogeosciences*, *20*(24), 5229–5242. https://doi.org/10.5194/bg-20-5229-2023

Houba, V. J. G., Temminghoff, E. J. M., Gaikhorst, G. A., & van Vark, W. (2000). Soil analysis procedures using 0.01 *M* calcium chloride as extraction reagent. *Communications in Soil Science and Plant Analysis*, *31*(9–10), 1299–1396. https://doi.org/10.1080/00103620009370514

Le Hir, P., Cayocca, F., & Waeles, B. (2011). Dynamics of sand and mud mixtures: A multiprocess-based modelling strategy. *Continental Shelf Research*, *31*(10 SUPPL.). https://doi.org/10.1016/j.csr.2010.12.009

Lorrain, A., Savoye, N., Chauvaud, L., Paulet, Y. M., & Naulet, N. (2003). Decarbonation and preservation method for the analysis of organic C and N contents and stable isotope ratios of low-carbonated suspended particulate material. *Analytica Chimica Acta*, *491*(2), 125–133. https://doi.org/10.1016/S0003-2670(03)00815-8

Robbins, J. A., & Edgington, D. N. (1975). Determination of recent sedimentation rates in Lake Michigan using Pb-210 and Cs-137. *Geochimica et Cosmochimica Acta*, *39*(3), 285–304. https://doi.org/10.1016/0016-7037(75)90198-2

Sebag, D., Verrecchia, E. P., Adatte, T., Aubert, M., Cailleau, G., Decaëns, T., Kowalewski, I., Trap, J., Bureau, F., & Hedde, M. (2022). Size fractions of organic matter pools influence their stability: Application of the Rock-Eval® analysis to beech forest soils. *Pedosphere*, *32*(4), 565–575. https://doi.org/10.1016/S1002-0160(21)60050-4

Shakeel, A., Zander, F., de Klerk, J. W., Kirichek, A., Gebert, J., & Chassagne, C. (2022). Effect of organic matter degradation in cohesive sediment: a detailed rheological analysis. *Journal of Soils and Sediments*, *22*(11), 2883–2892. https://doi.org/10.1007/S11368-022-03156-5/FIGURES/6

Supplementary tables

Table S1 Target genes, primer names and sequences, amplicon sizes, and references used for quantitative PCR. Total *pmoA* (*pmoAtot*) abundance was calculated by summing the gene copy numbers of the three *pmoA* clades, providing a comprehensive estimate of particulate methane monooxygenase gene abundance in the samples.

Target Gene	Primer Name	Sequence (5'→3')	Amplicon Size (bp)	Thermal Conditions	Reference
mcrA	mcrA_F_mod_SLB	GGTGTMGGATTCACMCARTAYGCWAC	500	Denaturation: 95°C/15min<br>- 39 cycles: 95°C/15s; 57°C/45s; 72°C/45s; 78°C/5s (data acquisition)<br>- Melting curve analysis: 0.5°C/0.05s from 65°C to 95°C	Modified by S. Brauer for detecting *M. boonei*, original Hales ME2; also modified by Luton et al. (2002)
	mcrA_R_mod_SLB	TTCATTGCRTAGTTHGGRTAGTT			
mmoX	mmoXLF	GAAGATTGGGGCGGCATCTG	427	Denaturation: 95°C/3min<br>- 39 cycles: 95°C/15s; 63°C/30s; 80°C/5s (data acquisition)<br>- Melting curve analysis: 0.5°C/0.05s from 65°C to 95°C	Rahman et al. (2011)
	mmoXLR	CCCAATCATCGCTGAAGGAGT			
pmoAIa	A189f	GGNGACTGGGACTTCTGG	491	Denaturation: 95°C/3min<br>- 39 cycles: 95°C/15s; 60°C/30s; 72°C/30s; 80°C/5s (data acquisition)<br>- Melting curve analysis: 0.5°C/0.05s from 65°C to 95°C	Kolb et al. (2003)
	mb601R	ACRTAGTGGTAACCTTGYAA			
pmoAIb	A189f	GGNGACTGGGACTTCTGG	491	Denaturation: 95°C/3min<br>- 39 cycles: 95°C/15s; 60°C/30s; 72°C/30s; 80°C/5s (data acquisition)<br>- Melting curve analysis: 0.5°C/0.05s from 65°C to 95°C	Kolb et al. (2003)
	mc468R	GCSGTGAACAGGTAGCTGCC			
pmoAII	II223F	CGTCGTATGTGGCCGAC	491	Denaturation: 95°C/3min<br>- 39 cycles: 95°C/15s; 67°C/30s; 72°C/30s; 82°C/5s (data acquisition)<br>- Melting curve analysis: 0.5°C/0.05s from 65°C to 95°C	Kolb et al. (2003)
	II646R	CGTGCCGCGCTCGACCATGYG			
**16S rRNA**	16S-341F	CCTACGGGNGGCWGCAG	464	Denaturation: 95°C/3min<br>- 39 cycles: 95°C/15s; 60°C/1min; 80°C/5s (data acquisition)<br>- Melting curve analysis: 0.5°C/0.05s from 65°C to 95°C	Klindworth et al. (2012)
	16S-805R	GACTACHVGGGTATCTAATCC			

Table S2 Summary of significant mixed linear models explaining methane (CH_4) fluxes across different variable subsets. Models within each subset are ranked and grouped according to fixed or random effects: site, sediment type (bare vs. seagrass), and subsite. The top-performing models within each group (i.e., those with the lowest AICc) are highlighted in bold. All models use CH_4 flux as the response variable, with predictors drawn from physical (a), gas-related (b), chemical (c), microbial (d-l), or combined (m) variable categories. Only models containing significant ($p < 0.05$) or marginally significant (# $p < 0.1$) predictors within each subset are presented. Variables included in each subset are listed at the top of their respective tables, n = 83. Models were fitted using linear models (LM) or linear mixed-effects models (LME), depending on the inclusion of random effects. For model selection purposes only, models without random effects (LMs) were refitted using generalized least squares (GLS), allowing them to be compared on a consistent likelihood basis with mixed-effects models. AICc values were then computed from these GLS formulations for model comparison across all models.

(a) Physical

Subset of physical variables: Clay, Sand, Immersion_time, Hydro_forcing, Bathymetry, DBD, Moisture					
**Model**	**Formula: Fixed part**	**Random part**	**AICc (GLS)**	**Fixed R^2**	**Global R^2**
**1**	**$\log_{10}(CH_4\_flux)$ = 0.21 x Sand**	**~1|Type**	**200**	**0.03**	**0.67**
**2**	**$\log_{10}(CH_4\_flux)$ = - 0.50 x DBD + 0.39 x Sand - 0.37 x $\log_{10}$(Moisture)**	**~1|Type**	**200**	**0.06**	**0.73**
**3**	**$\log_{10}(CH_4\_flux)$ = - 0.20 x $\log_{10}$(Moisture)**	**~1|Type**	**201**	**0.02**	**0.67**
**4**	**$\log_{10}(CH_4\_flux)$ = - 0.20 x Clay**	**~1|Type**	**201**	**0.02**	**0.67**
**5**	**$\log_{10}(CH_4\_flux)$ = - 1.74 x Hydro_forcing + 0.94 x Bathymetry - 0.78 x DBD**	**~1+DBD|Site**	**202**	**0.50**	**0.88**
6	$\log_{10}(CH_4\_flux)$ = - 1.87 x Hydro_forcing + 1.51 x Immersion_time - 0.48 x DBD	~1|Site	220	0.45	0.88
7	$\log_{10}(CH_4\_flux)$ = - 1.76 x Hydro_forcing + 1.12 x Bathymetry - 0.59 x DBD	~1|Site	223	0.45	0.86
**8**	**$\log_{10}(CH_4\_flux)$ = - 0.57 x Hydro_forcing - 0.46 x DBD**		**224 (232)**	**0.18**	

(b) Gas-related

Subset of Gas variables: CO_2_flux, CO_2_aerobic, CH_4_prod, CO_2_anaerobic, CH_4_oxi					
**Model**	**Formula: Fixed part**	**Random part**	**AICc (GLS)**	**Fixed R^2**	**Global R^2**
**1**	**$\log_{10}(CH_4\_flux)$ = 0.30 x CO_2_flux**	**~1|Type/Site**	**183**	**0.06**	**0.76**
**2**	**$\log_{10}(CH_4\_flux)$ = 0.35 x CO_2_flux**	**~1|Subsite**	**190**	**0.11**	**0.69**
**3**	**$\log_{10}(CH_4\_flux)$ = 0.25 x CO_2_flux**	**~1|Type**	**196**	**0.05**	**0.64**
**4**	**$\log_{10}(CH_4\_flux)$ = 0.40 x $\log_{10}(CO_2\_anaerobic)$**		**226 (232)**	**0.15**	
**5**	**$\log_{10}(CH_4\_flux)$ = 0.43 x $\log_{10}(CO_2\_anaerobic)$**	**~1|Site**	**233**	**0.18**	**0.22**

(c) Chemical

Subset of chemical variables: pH, Conductivity, N, NH_4, NO_2, NO_3, HI, C_{min}, C_{org}, CAR, SAR, MAR					
**Model**	**Formula: Fixed part**	**Random part**	**AICc (GLS)**	**Fixed R^2**	**Global R^2**
**1**	**$log_{10}(CH_4$_flux) = 0.48 x CAR**	**~1|Subsite**	**192**	**0.22**	**0.65**
**2**	**$log_{10}(CH_4$_flux) = 0.27 x CAR - 0.22 x HI**	**~1|Type**	**199**	**0.07**	**0.59**
3	$log_{10}(CH_4$_flux) = - 0.26 x HI + 0.24 x MAR	~1|Type	200	0.05	0.66
4	$log_{10}(CH_4$_flux) = 0.21 x CAR	~1|Type	201	0.04	0.55
**5**	**$log_{10}(CH_4$_flux) = - 0.24 x HI + 0.22 x SAR**	**~1|Type**	**201**	**0.05**	**0.62**
**6**	**$log_{10}(CH_4$_flux) = 0.61 x CAR**	**~1|Site**	**225**	**0.32**	**0.40**
**7**	**$log_{10}(CH_4$_flux) = 0.54 x CAR - 0.22 x HI**		**216 (225)**	**0.26**	
8	$log_{10}(CH_4$_flux) = 0.71 x C_{org} + 0.57 x SAR	~1|Site	225	0.40	0.70
9	$log_{10}(CH_4$_flux) = 0.65 x CAR - 0.24 x HI	~1|Site	226	0.34	0.41

(d) Microbial

Subset of microbial variables: *16S, mmoX, pmoA1a, pmoA1b, pmoAII, pmoAtot, mcrA,* DNA, pmoA_R, pmoA_D, mmoX_R, mmoX_D, mcrA_R, mcrA_D					
**Model**	**Formula: Fixed part**	**Random part**	**AICc (GLS)**	**Fixed R^2**	**Global R^2**
**1**	**$log_{10}(CH_4$_flux) = - 0.20 x mmoX_D + 0.19 x sqrt(pmoA_R)**	**~1|Type/Site**	**191**	**0.03**	**0.74**
**2**	**$log_{10}(CH_4$_flux) = 0.25 x sqrt(pmoA_R) - 0.24 x mmoX_D - 0.22 x log_{10}(DNA)**	**~1|Type**	**193**	**0.09**	**0.76**
3	$log_{10}(CH_4$_flux) = 0.30 x sqrt(pmoA_R) - 0.28 x mmoX_D	~1|Type	194	0.07	0.69
4	$log_{10}(CH_4$_flux) = - 0.30 x log_{10}(DNA)	~1|Type	195	0.05	0.73
5	$log_{10}(CH_4$_flux) = 0.29 x sqrt(pmoA_R) - 0.24 x mmoX_D + 0.17 x log_{10}(mcrA_D)	~1|Type	195	0.09	0.71
**6**	**$log_{10}(CH_4$_flux) = 0.41 x sqrt(pmoA_R)**		**226 (231)**	**0.16**	
**7**	**$log_{10}(CH_4$_flux) = 0.41 x sqrt(pmoA_R)**	**~1|Site**	**234**	**0.16**	**0.16**
8	$log_{10}(CH_4$_flux) = 0.40 x sqrt(pmoA_D) + 0.25 x log10(mcrA_D)		226 (235)	0.16	

(e) Microbial qPCR

Subset of microbial qPCR variables: *16S*, *mmoX*, *pmoA1a*, *pmoA1b*, *pmoAII*, *pmoAtot*, *mcrA*, DNA					
**Model**	**Formula: Fixed part**	**Random part**	**AICc (GLS)**	**Fixed R^2**	**Global R^2**
**1**	**$log_{10}(CH_4$_flux) = - 0.30 x log_{10}(DNA)**	**~1|Type**	**195**	**0.05**	**0.73**
**2**	**$log_{10}(CH_4$_flux) = 0.77 x log_{10}*(pmoAII)* - 0.73 x log_{10}*(pmoA1a)***		**236 (242)**	**0.06**	
**3**	**$log_{10}(CH_4$_flux) = 0.57 x log_{10}*(pmoAII)* - 0.55 x log_{10}*(mmoX)***		**236 (243)**	**0.06**	
**4**	**$log_{10}(CH_4$_flux) = 0.49 x log_{10}*(16S)* - 0.48 x log_{10}*(mmoX)***		**238 (244)**	**0.07**	
**5**	**$log_{10}(CH_4$_flux) = 0.77 x log_{10}*(pmoAII)* - 0.73 x log_{10}*(pmoA1a)***	**~1|Site**	**245**	**0.08**	**0.08**
**6**	**$log_{10}(CH_4$_flux) = 0.57 x log_{10}*(pmoAII)* - 0.55 x log_{10}*(mmoX)***	**~1|Site**	**245**	**0.08**	**0.08**
**7**	**$log_{10}(CH_4$_flux) = 0.51 x log_{10}*(16S)* - 0.50 x log_{10}*(mmoX)***	**~1|Site**	**246**	**0.07**	**0.09**

(f) Microbial diversity

Subset of microbial qPCR diversity: pmoA_R, pmoA_D, mmoX_R, mmoX_D ,mcrA_R, mcrA_D					
**Model**	**Formula: Fixed part**	**Random part**	**AICc (GLS)**	**Fixed R^2**	**Global R^2**
**1**	**$log_{10}(CH_4$_flux) = - 0.20 x mmoX_D + 0.19 x sqrt(pmoA_R)**	**~1|Type/Site**	**191**	**0.03**	**0.74**
**2**	**$log_{10}(CH_4$_flux) = 0.30 x sqrt(pmoA_R) - 0.28 x mmoX_D**	**~1|Type**	**194**	**0.07**	**0.70**
3	$log_{10}(CH_4$_flux) = - 0.28 x mmoX_D + 0.30 x sqrt(pmoA_R) + 0.17 x log_{10}(mcrA_D)	~1|Type	195	0.09	0.71
**4**	**$log_{10}(CH_4$_flux) = 0.41 x sqrt(pmoA_R)**		**226 (231)**	**0.16**	
**5**	**$log_{10}(CH_4$_flux) = 0.41 x sqrt(pmoA_R)**	**~1|Site**	**234**	**0.16**	**0.16**
6	$log_{10}(CH_4$_flux) = 0.41 x sqrt(pmoA_R) + 0.18 x log_{10}(mcrA_D)	~1|Site	235	0.19	0.19
7	$log_{10}(CH_4$_flux) = 0.40 x sqrt(pmoA_D) + 0.25 x log_{10}(mcrA_D)		226 (235)	0.16	

(g) mcrA

Subset of microbial *mcrA* variables: *16S*, *mcrA*, DNA, mcrA_R, mcrA_D					
**Model**	**Formula: Fixed part**	**Random part**	**AICc (GLS)**	**Fixed R^2**	**Global R^2**
**1**	**$log_{10}(CH_4$_flux) = - 0.30 x log_{10}(DNA)**	**~1|Type**	**194**	**0.05**	**0.73**
**2**	**$log_{10}(CH_4$_flux) = - 0.27 x log_{10}(DNA) + 0.16 x log_{10}(mcrA_D)**	**~1|Type**	**196**	**0.06**	**0.74**

(h) mmoX

Subset of microbial *mmoX* variables: *16S*, *mmoX*, DNA, mmoX_R, mmoX_D					
**Model**	**Formula: Fixed part**	**Random part**	**AICc (GLS)**	**Fixed R^2**	**Global R^2**
**1**	**$\log_{10}(CH_4\_flux) = -0.30 \times \log_{10}(DNA)$**	**~1|Type**	**194**	**0.05**	**0.73**
**2**	**$\log_{10}(CH_4\_flux) = 0.49 \times \log_{10}(\mathit{16S}) - 0.48 \times \log_{10}(\mathit{mmoX})$**		**238 (244)**	**0.04**	
**3**	**$\log_{10}(CH_4\_flux) = 0.51 \times \log_{10}(\mathit{16S}) - 0.50 \times \log_{10}(\mathit{mmoX})$**	**~1|Site**	**246**	**0.07**	**0.09**

(i) pmoA

Subset of microbial *pmoA* variables: *16S*, *pmoA1a*, *pmoA1b*, *pmoAII*, *pmoAtot*, DNA, pmoA_R, pmoA_D					
**Model**	**Formula: Fixed part**	**Random part**	**AICc (GLS)**	**Fixed R^2**	**Global R^2**
**1**	**$\log_{10}(CH_4\_flux) = -0.30 \times \log_{10}(DNA)$**	**~1|Type**	**194**	**0.05**	**0.73**
**2**	**$\log_{10}(CH_4\_flux) = -0.26 \times \log_{10}(DNA) + 0.18 \times \log_{10}(pmoA\_R)$**	**~1|Type**	**195**	**0.07**	**0.71**
**3**	**$\log_{10}(CH_4\_flux) = 0.41 \times sqrt(pmoA\_R)$**		**226 (231)**	**0.16**	
**4**	**$\log_{10}(CH_4\_flux) = 0.41 \times sqrt(pmoA\_R)$**	**~1|Site**	**234**	**0.16**	**0.16**

(j) mcrA genus

Subset of *mcrA* genus variables: *Methermicoccus, Methanococcoides, Methanolobus, Methanogenium, Methanosarcina, Methanobacterium, Methanoculleus, Methanocalculus, Methanolinea, Methanopyrus, Methanocaldococcus, Methanococcus_fervidus, Methanohalophilus, Methanocella*					
**Model**	**Formula: Fixed part**	**Random part**	**AICc (GLS)**	**Fixed R^2**	**Global R^2**
**1**	**$log_{10}(CH_4$_flux) = 0.21 x *Methanopyrus***	**~1|Type**	**200**	**0.03**	**0.65**
**2**	**$log_{10}(CH_4$_flux) = 0.17 x *Methanocaldococcus***	**~1|Type**	**202**	**0.02**	**0.61**
**3**	**$log_{10}(CH_4$_flux) = 0.32 x *Methanolobus* + 0.24 x *Methanocaldococcus* - 0.23 x *Methanosarcina***		**230 (241)**	**0.14**	
**4**	**$log_{10}(CH_4$_flux) = 0.29 x *Methanolobus* - 0.25 x *Methanosarcina***		**233 (241)**	**0.09**	
**5**	**$log_{10}(CH_4$_flux) = 0.26 x *Methanolobus* + 0.25 x *Methanocaldococcus***		**233 (241)**	**0.09**	
**6**	**$log_{10}(CH_4$_flux) = - 0.22 x *Methanolinea***		**237 (242)**	**0.04**	
**7**	**$log_{10}(CH_4$_flux) = 0.23 x *Methanolobus***		**236 (242)**	**0.04**	
**8**	**$log_{10}(CH_4$_flux) = 0.30 x *Methanolobus* - 0.28 x *Methanosarcina* - 0.25 x *Methanolinea* + 0.23 x *Methanocaldococcus***	**~1|Site**	**243**	**0.22**	**0.30**
**9**	**$log_{10}(CH_4$_flux) = - 0.29 x *Methanosarcina* + 0.28 x *Methanolobus* - 0.26 x *Methanolinea***	**~1|Site**	**243**	**0.18**	**0.25**
**10**	**$log_{10}(CH_4$_flux) = 0.32 x *Methanolobus* - 0.25 x *Methanosarcina* + 0.24 x *Methanocaldococcus***	**~1|Site**	**243**	**0.17**	**0.19**
**11**	**$log_{10}(CH_4$_flux) = - 0.28 x *Methanolinea* - 0.24 x *Methanosarcina***	**~1|Site**	**245**	**0.13**	**0.20**
**12**	**$log_{10}(CH_4$_flux) = - 0.25 x *Methanolinea* + 0.23 x *Methanocaldococcus***	**~1|Site**	**245**	**0.11**	**0.16**
**13**	**$log_{10}(CH_4$_flux) = 0.29 x *Methanolobus* - 0.26 x *Methanosarcina***	**~1|Site**	**243**	**0.12**	**0.13**
**14**	**$log_{10}(CH_4$_flux) = 0.27 x *Methanolobus* + 0.25 x *Methanocaldococcus***	**~1|Site**	**243**	**0.11**	**0.11**
**15**	**$log_{10}(CH_4$_flux) = - 0.24 x *Methanolinea***	**~1|Site**	**244**	**0.06**	**0.08**
**16**	**$log_{10}(CH_4$_flux) = 0.23 x *Methanolobus***	**~1|Site**	**244**	**0.05**	**0.05**
**17**	**$log_{10}(CH_4$_flux) = 0.22 x *Methanolobus* - 0.22 x *Methanolinea***	**~1|Site**	**245**	**0.10**	**0.12**
**18**	**$log_{10}(CH_4$_flux) = 0.25 x *Methanolobus* + 0.25 x *Methanocaldococcus* - 0.21 x *Methanolinea***	**~1|Site**	**245**	**0.16**	**0.19**

(k) mmoX genus

Subset of *mmoX* genus variables: *Methylocella, Methyloferula, Methylocystis_m, Methylosinus_m, Methylomicrobium_m, Methylovulum_m, Methylomonas_m, Methylococcus_m, Methylomagnum_m, Methylocaldum_m, Pseudomonas, Mycobacterium, Rhodococcus, Methylocella_prmA, Methylibium, Bradyrhizobium, Paracoccus, Gordonia*					
**Model**	**Formula: Fixed part**	**Random part**	**AICc (GLS)**	**Fixed R^2**	**Global R^2**
**1**	**log10(CH4_flux) = - 0.18 x *Methyloferula***	**~1|Type/Site**	**186**	**0.02**	**0.76**
**2**	**log10(CH4_flux) = - 0.19 x *Methyloferula***	**~1|Subsite**	**193**	**0.03**	**0.68**
**3**	**log10(CH4_flux) = 0.27 x *Methylomicrobium_m* - 0.24 x *Methylomonas_m* + 0.20 x *Methylocella* - 0.16 x *Methyloferula***	**~1|Type**	**209**	**0.06**	**0.66**
**4**	**log10(CH4_flux) = - 0.43 x *Methylomagnum_m* + 0.37 x *Methylocaldum_m***		**237 (244)**	**0.05**	
**5**	**log10(CH4_flux) = - 0.43 x *Methylomagnum_m* + 0.37 x *Methylocaldum_m***	**~1|Site**	**246**	**0.07**	**0.07**

(l) pmoA genus

Subset of *pmoA* genus variables: *Methylocaldum_p, Methylogaea, Methylosinus_p, Methylocystis_p, Methylocapsa, Methylomonas_p, Methylomagnum_p, Candidatus_Binatia, Candidatus_Methylomirabilis, Methylomarinum, Methylomicrobium_p, Methyloglobulus, Methylovulum_p, Methylosoma, Methylobacter, Methylococcus_p, Clonothrix*					
**Model**	**Formula: Fixed part**	**Random part**	**AICc (GLS)**	**Fixed R^2**	**Global R^2**
**1**	**$log_{10}(CH_4\_flux)$ = 0.17 x *Methylogaea***	**~1|Subsite**	**194**	**0.03**	**0.69**
2	$log_{10}(CH_4\_flux)$ = 0.27 x *Methyloglobulus* + 0.24 x *Methylococcus_p*		232 (240)	0.10	
**3**	**$log_{10}(CH_4\_flux)$ = 0.28 x *Methyloglobulus* + 0.22 x *Methylococcus_p* + 0.21 x *Methylomarinum***		**230 (241)**	**0.14**	
**4**	**$log_{10}(CH_4\_flux)$ = 0.26 x *Methyloglobulus***	**~1|Site**	**242**	**0.07**	**0.07**
5	$log_{10}(CH_4\_flux)$ = 0.27 x *Methyloglobulus* + 0.24 x *Methylococcus_p*	~1|Site	242	0.12	0.12
6	$log_{10}(CH_4\_flux)$ = 0.28 x *Methyloglobulus* + 0.22 x *Methylococcus_p* + 0.21 x *Methylomarinum*	~1|Site	243	0.16	0.16
7	$log_{10}(CH_4\_flux)$ = 0.27 x *Methyloglobulus* + 0.23 x *Methylomarinum*	~1|Site	243	0.12	0.12
8	$log_{10}(CH_4\_flux)$ = 0.22 x *Methylococcus_p*	~1|Site	244	0.05	0.05
9	$log_{10}(CH_4\_flux)$ = 0.22 x *Methylomarinum*	~1|Site	244	0.05	0.05

(m) Combined

Subset of combined variables: CO2_flux, CO2_anaerobic, CAR, HI, MAR, SAR, DBD, Sand, Hydro_forcing, Bathymetry, pmoA_R, DNA, mmoX_D, *pmoAII*, *16S*, pmoA_D, *Methyloferula, Methylogaea, Methanopyrus or Methanosarcina, Methanocaldococcus, Methyloglobulus, Methylomarinum, Methylomicrobium_m, Methylomonas_m, Methylocella, Methylococcus_p, Methanolobus, Methanolinea, Methylomagnum_m, Methylocaldum_m*					
Model	**Formula: Fixed part**	**Random part**	**AICc (GLS)**	**Fixed R^2**	**Global R^2**
1	$\log_{10}(CH_4\_flux) = 0.33 \times CO_2\_flux + 0.19 \times$ *Methylogaea*	~1|Type/Site	182	0.09	0.80
2	$\log_{10}(CH_4\_flux) = 0.32 \times CO_2\_flux + 0.18 \times$ *Methylogaea* - 0.16 x *Methyloferula*	~1|Type/Site	182	0.11	0.82
3	$\log_{10}(CH_4\_flux) = 0.32 \times CO_2\_flux + 0.31 \times CAR - 0.21 \times HI + 0.20 \times$ *Methylogaea*	~1|Type/Site	182	0.17	0.75
4	**$\log_{10}(CH_4\_flux) = 0.30 \times CO_2\_flux$**	**~1|Type/Site**	**183**	**0.06**	**0.76**
5	$\log_{10}(CH_4\_flux) = 0.30 \times CO_2\_flux - 0.17 \times$ *Methyloferula*	~1|Type/Site	183	0.09	0.79
6	$\log_{10}(CH_4\_flux) = 0.31 \times CO_2\_flux + 0.20 \times$ *Methylogaea* - 0.18 x HI	~1|Type/Site	183	0.09	0.81
7	$\log_{10}(CH_4\_flux) = 0.32 \times CO_2\_flux + 0.32 \times CAR - 0.23 \times HI + 0.20 \times$ *Methylogaea* + 0.14 x *Methylomicrobium_m*	~1|Type/Site	184	0.20	0.75
8	$\log_{10}(CH_4\_flux) = 0.53 \times CAR + 0.37 \times CO_2\_flux - 0.17 \times$ *Methyloferula* + 0.17 x *Methylogaea*	~1|Subsite	186	0.37	0.76
9	$\log_{10}(CH_4\_flux) = 0.52 \times CAR + 0.39 \times CO_2\_flux + 0.18 \times$ *Methylogaea*	~1|Subsite	186	0.35	0.73
10	$\log_{10}(CH_4\_flux) = 0.53 \times CAR + 0.35 \times CO_2\_flux - 0.18 \times$ *Methyloferula*	~1|Subsite	186	0.36	0.72
11	$\log_{10}(CH_4\_flux) = 0.53 \times CAR + 0.37 \times CO_2\_flux - 0.17 \times$ *Methyloferula* + 0.17 x *Methylogaea* + 0.13 x *Methylomicrobium_m*	~1|Subsite	187	0.39	0.77
12	$\log_{10}(CH_4\_flux) = 0.57 \times CAR + 0.36 \times CO_2\_flux + 0.19 \times$ *Methylogaea* - 0.18 x HI	~1|Subsite	187	0.37	0.73
13	**$\log_{10}(CH_4\_flux) = 0.52 \times CAR + 0.36 \times CO_2\_flux$**	**~1|Subsite**	**187**	**0.34**	**0.69**
14	$\log_{10}(CH_4\_flux) = 0.32 \times CAR + 0.31 \times CO_2\_flux - 0.27 \times HI + 0.18 \times$ *Methylogaea*	~1|Type	188	0.17	0.68
15	$\log_{10}(CH_4\_flux) = 0.31 \times CAR + 0.26 \times CO_2\_flux - 0.23 \times HI - 0.19 \times \log_{10}(DNA) + 0.18 \times$ *Methylogaea*	~1|Type	189	0.16	0.74

16	$\log_{10}$(CH_4_flux) = 0.33 x CAR + 0.32 x CO_2_flux - 0.29 x HI + 0.18 x *Methylogaea* + 0.16 x *Methylomicrobium_m*	~1|Type	189	0.21	0.68
**17**	**$\log_{10}$(CH_4_flux) = 0.33 x CAR + 0.30 x CO_2_flux - 0.24 x HI**	**~1|Type**	**189**	**0.17**	**0.63**
18	$\log_{10}$(CH_4_flux) = 0.32 x CAR + 0.25 x CO_2_flux - 0.20 x HI - 0.20 x $\log_{10}$(DNA)	~1|Type	189	0.15	0.70
19	$\log_{10}$(CH_4_flux) = 0.34 x CAR + 0.25 x CO_2_flux - 0.22 x HI - 0.19 x $\log_{10}$(DNA) + 0.15 x *Methylomicrobium_m*	~1|Type	190	0.18	0.70
20	$\log_{10}$(CH_4_flux) = 0.32 x CAR + 0.30 x CO_2_flux - 0.27 x HI + 0.18 x *Methylogaea* + 0.15 x *Methylomarinum*	~1|Type	190	0.20	0.69
21	$\log_{10}$(CH_4_flux) = 0.27 x CAR + 0.23 x CO_2_flux - 0.25 x $\log_{10}$(DNA)	~1|Type	190	0.12	0.69
22	$\log_{10}$(CH_4_flux) = 0.32 x CO_2_flux + 0.30 x CAR - 0.30 x $\log_{10}$(DNA) - 0.30 x DBD	~1|Type	190	0.15	0.68
23	$\log_{10}$(CH_4_flux) = 0.35 x CAR + 0.31 x CO_2_flux - 0.26 x HI + 0.16 x *Methylomicrobium_m*	~1|Type	190	0.20	0.64
24	$\log_{10}$(CH_4_flux) = 0.80 x CAR + 0.57 x CO_2_flux + 0.19 x *Methylomicrobium_m* + 0.19 x *Methylomarinum*	~1|Site	212	0.55	0.73
25	$\log_{10}$(CH_4_flux) = 0.78 x CAR + 0.52 x CO_2_flux + 0.19 x *Methylomicrobium_m* + 0.18 x *Methanocaldococcus* + 0.18 x *Methylomarinum*	~1|Site	212	0.56	0.73
26	$\log_{10}$(CH_4_flux) = 0.86 x CAR + 0.61 x CO_2_flux + 0.32 x Bathymetry + 0.18 x *Methylomarinum*	~1|Site	212	0.53	0.72
27	$\log_{10}$(CH_4_flux) = 0.79 x CAR + 0.51 x CO_2_flux + 0.19 x *Methylomicrobium_m* + 0.18 x *Methanocaldococcus*	~1|Site	212	0.54	0.70
**28**	**$\log_{10}$(CH_4_flux) = 0.82 x CAR + 0.58 x CO_2_flux**	**~1|Site**	**212**	**0.51**	**0.69**
29	$\log_{10}$(CH_4_flux) = 0.74 x CAR + 0.54 x CO_2_flux - 0.19 x *Methanolinea* + 0.18 x *Methylomicrobium_m*	~1|Site	212	0.54	0.67
30	$\log_{10}$(CH_4_flux) = 0.81 x CAR + 0.57 x CO_2_flux + 0.19 x *Methylomicrobium_m*	~1|Site	212	0.53	0.71

31	$\log_{10}(CH_4\_flux)$ = 0.81 x CAR + 0.58 x CO_2_flux + 0.18 x *Methylomarinum*	~1|Site	212	0.53	0.71
32	$\log_{10}(CH_4\_flux)$ = 0.73 x CAR + 0.55 x CO_2_flux - 0.20 x *Methanolinea* + 0.18 x *Methylomarinum*	~1|Site	212	0.54	0.68
33	$\log_{10}(CH_4\_flux)$ = 0.78 x CAR + 0.49 x CO_2_flux - 0.22 x HI + 0.21 x *Methylomicrobium_m* + 0.19 x *Methylomarinum*	~1|Site	212	0.56	0.67
34	$\log_{10}(CH_4\_flux)$ = 0.74 x CAR + 0.54 x CO_2_flux - 0.21 x *Methanolinea*	~1|Site	212	0.52	0.65
35	**$\log_{10}(CH_4\_flux)$ = 0.64 x CAR + 0.46 x CO_2_flux - 0.25 x HI - 0.24 x Sand + 0.22 x *Methylomicrobium_m***		**193 (212)**	**0.47**	
36	$\log_{10}(CH_4\_flux)$ = 0.96 x CAR + 0.59 x CO_2_flux - 0.19 x DNA + 0.19 x *Methylomicrobium_m*	~1|Site	213	0.55	0.78
37	$\log_{10}(CH_4\_flux)$ = 0.92 x CAR + 0.57 x CO_2_flux + 0.37 x Bathymetry + 0.18 x *Methylomicrobium_m* - 0.18 x *Methyloferula*	~1|Site	213	0.54	0.77
38	$\log_{10}(CH_4\_flux)$ = 0.93 x CAR + 0.58 x CO_2_flux + 0.38 x Bathymetry - 0.18 x *Methyloferula*	~1|Site	213	0.52	0.75
39	$\log_{10}(CH_4\_flux)$ = 0.78 x CAR + 0.53 x CO_2_flux + 0.18 x *Methanocaldococcus* + 0.18 x *Methylomarinum*	~1|Site	213	0.54	0.71
40	$\log_{10}(CH_4\_flux)$ = 0.73 x CAR + 0.54 x CO_2_flux - 0.19 x *Methanolinea* + 0.18 x *Methylomarinum* + 0.18 x *Methylomicrobium_m*	~1|Site	213	0.56	0.70
41	$\log_{10}(CH_4\_flux)$ = 0.79 x CAR + 0.52 x CO_2_flux + 0.18 x *Methanocaldococcus*	~1|Site	213	0.52	0.68
42	$\log_{10}(CH_4\_flux)$ = 0.72 x CAR + 0.50 x CO_2_flux - 0.20 x *Methanolinea* + 0.18 x *Methanocaldococcus* + 0.18 x *Methylomarinum*	~1|Site	213	0.55	0.68
43	$\log_{10}(CH_4\_flux)$ = 0.72 x CAR + 0.49 x CO_2_flux - 0.19 x *Methanolinea* + 0.18 x *Methanocaldococcus* + 0.18 x *Methylomicrobium_m*	~1|Site	213	0.55	0.67
44	$\log_{10}(CH_4\_flux)$ = 0.72 x CAR + 0.49 x CO_2_flux - 0.20 x *Methanolinea* + 0.18 x *Methanocaldococcus*	~1|Site	213	0.53	0.65
45	$\log_{10}(CH_4\_flux)$ = 0.79 x CAR + 0.50 x CO_2_flux - 0.22 x HI + 0.21 x *Methylomicrobium_m*	~1|Site	213	0.53	0.65
46	$\log_{10}(CH_4\_flux)$ = 0.60 x CAR + 0.34 x CO_2_flux - 0.29 x HI + 0.23 x *Methylomicrobium_m* + 0.20 x *Methylomarinum*		194 (213)	0.46	
47	$\log_{10}(CH_4\_flux)$ = 1.01 x CAR + 0.56 x CO_2_flux - 0.23 x DNA + 0.19 x *Methylomicrobium_m* - 0.19 x *Methyloferula*	~1|Site	214	0.56	0.80
48	$\log_{10}(CH_4\_flux)$ = 0.69 x CAR + 0.56 x CO_2_flux - 0.25 x Sand	~1|Site	214	0.47	0.56

Table S3 Microbial characteristics of the studied sites. (a) DNA content and qPCR results. (b) Diversity indices. Values are expressed as mean ± standard error (SE), n = 83. Sites are ordered along a gradient from the most negative to the most positive values along PC1 (see Figure 2), which reflects a multivariate ordination of environmental and microbial descriptors. Differences among sites were assessed using non-parametric Kruskal–Wallis tests, followed by Dunn's post hoc tests with Benjamini–Hochberg correction for multiple comparisons. Different letters indicate significant differences among sites for each variable ($p < 0.05$); groups sharing at least one letter are not significantly different.

(a)

**Site_ID**	**DNA ng m^{-2}**		***16S* copies m^{-2}**		***mcrA* copies m^{-2}**		***mmoX* copies m^{-2}**		***pmoA1a* copies m^{-2}**		***pmoA1b* copies m^{-2}**		***pmoAII* copies m^{-2}**		***pmoAtot* copies m^{-2}**	
Gail	1.5e+09 ± 2.5e+08	bc	6.2e+13 ± 4.5e+12	d	1.3e+10 ± 3.6e+09	b	5.4e+09 ± 4.8e+08	c	2.9e+10 ± 2.3e+09	c	4.1e+11 ± 3.1e+10	d	2.3e+10 ± 2.8e+09	c	4.6e+11 ± 3.5e+10	d
Garr	8.6e+08 ± 2.1e+08	ab	6.7e+13 ± 1.3e+13	d	2.0e+10 ± 4.2e+09	ab	5.5e+09 ± 9.7e+08	c	3.1e+10 ± 5.1e+09	c	4.6e+11 ± 7.9e+10	d	3.3e+10 ± 9.7e+09	c	5.2e+11 ± 9.2e+10	d
Jacq	1.7e+09 ± 5.1e+08	abc	9.5e+13 ± 8.5e+12	cd	1.1e+10 ± 2.2e+09	b	7.2e+09 ± 6.4e+08	bc	4.0e+10 ± 3.0e+09	bc	5.9e+11 ± 4.2e+10	cd	4.1e+10 ± 3.9e+09	bc	6.7e+11 ± 4.8e+10	cd
Germ	1.5e+09 ± 1.7e+08	c	1.4e+14 ± 2.4e+13	bcd	1.6e+10 ± 3.2e+09	ab	1.3e+10 ± 1.8e+09	ab	7.7e+10 ± 1.2e+10	ab	9.3e+11 ± 1.3e+11	ac	7.2e+10 ± 1.1e+10	ab	1.1e+12 ± 1.5e+11	ac
Comp	8.9e+08 ± 1.5e+08	abc	1.7e+14 ± 1.5e+13	abc	1.4e+10 ± 4.3e+09	b	1.2e+10 ± 1.2e+09	ab	7.1e+10 ± 6.9e+09	ab	8.5e+11 ± 7.9e+10	ac	7.0e+10 ± 9.4e+09	ab	9.9e+11 ± 9.4e+10	ac
Ande	1.0e+09 ± 1.1e+08	abc	1.9e+14 ± 2.1e+13	ab	3.8e+10 ± 7.9e+09	a	2.1e+10 ± 2.4e+09	a	8.5e+10 ± 6.4e+09	a	1.4e+12 ± 1.0e+11	ab	1.0e+11 ± 8.9e+09	a	1.6e+12 ± 1.1e+11	ab
Font	6.6e+08 ± 5.5e+07	a	3.1e+14 ± 3.5e+13	a	3.4e+10 ± 6.1e+09	a	2.4e+10 ± 3.3e+09	a	1.2e+11 ± 1.5e+10	a	1.8e+12 ± 1.8e+11	b	1.1e+11 ± 1.6e+10	a	2.0e+12 ± 2.0e+11	b
All	1.2e+09 ± 1.0e+08		1.5e+14 ± 1.1e+13		2.1e+10 ± 2.1e+09		1.2e+10 ± 1.0e+09		6.5e+10 ± 4.7e+09		9.2e+11 ± 6.4e+10		6.5e+10 ± 5.0e+09		1.0e+12 ± 7.3e+10	

(b)

Site_ID	mcrA_R		mcrA_D		mmoX_R		mmoX_D		pmoA_R		pmoA_D	
Gail	50.2 ± 8.3	bc	3.1 ± 0.1	ab	27.1 ± 1.5	b	2.9 ± 0.1	ab	5.4 ± 0.8	c	0.5 ± 0.1	b
Garr	53.5 ± 3.6	c	3.5 ± 0.1	b	28.6 ± 1.7	ab	2.7 ± 0.1	b	8.1 ± 0.9	bc	0.4 ± 0.1	b
Jacq	39.1 ± 10.5	a	2.9 ± 0.2	a	28.0 ± 1.6	b	2.7 ± 0.1	b	6.8 ± 0.8	bc	0.4 ± 0.1	b
Germ	24.9 ± 2.0	a	2.7 ± 0.1	a	29.8 ± 1.7	ab	3.0 ± 0.0	ab	7.2 ± 0.5	bc	0.6 ± 0.1	ab
Comp	26.6 ± 2.1	a	2.8 ± 0.1	a	35.4 ± 1.3	a	3.1 ± 0.0	a	9.5 ± 0.8	ab	0.5 ± 0.1	b
Ande	31.0 ± 2.8	ab	2.8 ± 0.1	a	30.4 ± 1.5	ab	3.0 ± 0.0	ab	14.5 ± 1.5	a	1.1 ± 0.1	a
Font	27.8 ± 3.1	a	2.7 ± 0.1	a	24.8 ± 2.0	b	2.8 ± 0.1	b	10.2 ± 2.1	abc	0.8 ± 0.2	ab
All	36.3 ± 2.4		2.9 ± 0.0		29.1 ± 0.7		2.9 ± 0.0		8.8 ± 0.5		0.6 ± 0.0	

Table S4 Characteristics of the studied sites under bare and vegetated (seagrass) conditions, with variables grouped into general and physical features (a–b) and chemical variables (c–d). Values are reported as mean ± standard error (SE). The dataset includes a total of 83 observations, comprising 41 bare and 42 vegetated (seagrass) samples. Within each site, subsites represent spatially distinct sampling locations, each based on six replicate measurements, providing a hierarchical structure with measurements nested within subsites and subsites nested within sites. Significant differences (Wilcoxon tests) between bare and vegetated sediments are indicated as follows: # ($p < 0.1$), * ($p < 0.05$), ** ($p < 0.01$)), *** ($p < 0.001$), and NS (not significant), n = 83.

(a)

Site	Site_ID	Type (Subsite)	Longitude °E. WEGS84	Latitude °N. WGS84	Plant_cover		Sediment texture	Clay %		Silt %		Sand %	
Comprian	Comp	Bare	-1.070611	44.679473	0		Silty sand	3.0		47.4		49.6	
		Seagrass	-1.072061	44.679063	0.9		Silty sand	3.8		47.8		48.4	
Jacquets	Jacq	Bare	-1.18081	44.72352	0		Silty sand	1.8		29.5		68.7	
		Seagrass	-1.18128	44.72466	0.9		Silty sand	1.8		45.2		53.0	
Germanan	Germ	Bare	-1.13384771347	44.7116661072	0		Sandy silt	4.1		63.4		32.5	
		Seagrass	-1.13384771347	44.7116661072	0.9		Sandy silt	4.1		55.4		40.5	
Fontaines	Font	Bare	-1.07578	44.71885	0		Sand	0.6		8.4		91.0	
		Seagrass	-1.0754	44.71874	0.9		Sandy silt	3.6		52.4		44.0	
Garrèche	Garr	Bare	-1.1205807	44.7047449	0		Silty sand	1.7		26		72.3	
		Seagrass	-1.1208349	44.7040884	0.4		Silty sand	2.5		37.8		59.7	
Gaillard	Gail	Bare	-1.0994219	44.6627752	0		Sandy silt	3.5		61.2		35.3	
		Seagrass	-1.09981	44.66241	0.9		Sandy silt	3.2		60.4		36.4	
Andernos	Ande	Bare	-1.12038	44.74512	0		Sand	1.3		23.7		75.0	
		Seagrass	-1.12138	44.74513	0.9		Sand	1.3		21.5		77.2	
All sites	All	Bare	NA	NA	0.0	***	NA	2.3 ± 0.2	*	37.1 ± 3.2	NS	60.9 ± 3.2	NS
		Seagrass			0.8 ± 0.1			2.9 ± 0.2		45.8 ± 2.0		51.3 ± 2.0	

(b)

Site	Site_ID	Type (Subsite)	Bathymetry m		Hydrodynamic forcing		Immersion_time h day^{-1}		DBD g cm^{-3}		Moisture %	
Comprian	Comp	Bare	1.4		2.1e+05		18.3		1.1		39.3 ± 3.0	**
		Seagrass	-0.2		8.3e+04		10.6		1.0		55.0 ± 3.4	
Jacquets	Jacq	Bare	2.1		2.0e+05		18.1		1.2		36.3 ± 3.8	**
		Seagrass	0.6		1.4e+05		12.2		0.9		80.4 ± 5.0	
Germanan	Germ	Bare	1.7		1.3e+05		18.0		0.8		62.8 ± 3.5	NS
		Seagrass	1.7		1.3e+05		18.0		1.2		58.1 ± 4.5	
Fontaines	Font	Bare	-1.1		0		1.7		1.7		23.2 ± 0.5	**
		Seagrass	-1.1		0		1.7		1.2		38.5 ± 2.3	
Garrèche	Garr	Bare	1.2		2.3e+05		17.3		1.1		52.7 ± 5.3	NS
		Seagrass	1.1		1.1e+05		17.3		1.1		56.7 ± 7.3	
Gaillard	Gail	Bare	0.3		3.9e+05		13.1		0.8		79.8 ± 3.6	NS
		Seagrass	0.5		2.8e+05		13.1		0.8		74.0 ± 5.5	
Andernos	Ande	Bare	-0.8		0		1.7		1.4		34.8 ± 1.3	**
		Seagrass	-0.5		0		5.6		1.1		52.1 ± 2.7	
All sites	All	Bare	0.7 ± 0.2	#	1.6e+05 ± 2.0e+04	*	12.5 ± 1.1	NS	1.2 ± 0.0	#	47.2 ± 3.1	**
		Seagrass	0.3 ± 0.1		1.1e+05 ± 1.4e+04		11.2 ± 0.9		1.0 ± 0.0		59.2 ± 2.6	

(c)

Site_ID	Type (Subsite)	pH		Conductivity mS cm^{-1}		N %		NH_4^+ µg N-NH_4 g^{-1} dry soil		NO_2^- µg N-NO_2 g^{-1} dry soil		NO_3^- µg N-NO_3 g^{-1} dry soil	
Comp	Bare	8.5 ± 0.0	NS	3.5 ± 0.3	NS	0.070 ± 0.008	**	19.7 ± 5.3	NS	0.020 ± 0.003	NS	0.450 ± 0.213	NS
	Seagrass	8.5 ± 0.0		3.2 ± 0.2		0.119 ± 0.011		10.6 ± 2.1		0.019 ± 0.001		0.587 ± 0.088	
Jacq	Bare	8.6 ± 0.1	*	4.3 ± 0.4	*	0.068 ± 0.010	**	6.4 ± 0.6	*	0.017 ± 0.001	NS	0.409 ± 0.095	NS
	Seagrass	8.3 ± 0.1		6.9 ± 0.8		0.169 ± 0.007		9.7 ± 0.9		0.014 ± 0.001		0.534 ± 0.140	
Germ	Bare	7.3 ± 0.6	NS	6.6 ± 0.5	NS	0.139 ± 0.009	NS	6.6 ± 1.0	#	0.014 ± 0.003	NS	0.105 ± 0.101	#
	Seagrass	8.1 ± 0.2		6.0 ± 0.8		0.129 ± 0.014		8.1 ± 0.5		0.015 ± 0.002		0.439 ± 0.122	
Font	Bare	6.8 ± 0.1	**	4.4 ± 1.2	#	0.024 ± 0.008	**	4.3 ± 0.8	#	0.009 ± 0.001	**	0.219 ± 0.031	NS
	Seagrass	8.4 ± 0.1		4.3 ± 0.3		0.067 ± 0.011		6.8 ± 1.3		0.017 ± 0.002		0.163 ± 0.017	
Garr	Bare	8.4 ± 0.1	NS	5.9 ± 0.6	NS	0.116 ± 0.009	NS	5.9 ± 0.4	NS	0.020 ± 0.002	NS	0.253 ± 0.160	NS
	Seagrass	8.4 ± 0.1		6.4 ± 0.6		0.118 ± 0.009		6.3 ± 0.7		0.021 ± 0.010		0.431 ± 0.208	
Gail	Bare	8.0 ± 0.0	NS	7.4 ± 1.0	NS	0.202 ± 0.009	#	2.5 ± 0.4	#	0.016 ± 0.001	NS	0.072 ± 0.014	NS
	Seagrass	7.4 ± 0.4		6.6 ± 1.0		0.158 ± 0.017		5.0 ± 0.8		0.018 ± 0.001		0.152 ± 0.081	
Ande	Bare	6.8 ± 0.5	*	4.0 ± 0.2	**	0.155 ± 0.084	#	2.8 ± 0.8	*	0.020 ± 0.009	NS	0.064 ± 0.016	NS
	Seagrass	7.8 ± 0.1		6.2 ± 0.4		0.107 ± 0.015		5.4 ± 1.1		0.017 ± 0.001		0.082 ± 0.010	
All	Bare	7.8 ± 0.2	NS	5.2 ± 0.3	NS	0.111 ± 0.015	*	6.6 ± 1.0	**	0.016 ± 0.002	NS	0.219 ± 0.044	*
	Seagrass	8.1 ± 0.1		5.7 ± 0.3		0.124 ± 0.007		7.4 ± 0.5		0.017 ± 0.001		0.341 ± 0.050	

(d)

Site_ID	Type (Subsite)	$C_{mineral}$ %		$C_{organic}$ %		HI		SAR cm y^{-1}		MAR g cm^{-2} y^{-1}		CAR g CO_2-eq m^{-2} y^{-1}		Climate regulation g CO_2-eq m^{-2} y^{-1}	
Comp	Bare	0.082 ± 0.018	*	1.1		190.6 ± 3.0	NS	0.09		0.10		33.8		21.6 ± 2.8	**
	Seagrass	0.360 ± 0.095		0.6		201.0 ± 5.2		0.09		0.06		12.6		5.1 ± 1.1	
Jacq	Bare	0.580 ± 0.055	*	1.7		210.3 ± 6.7	*	0.23		0.33		64.4		62.3 ± 0.2	**
	Seagrass	0.267 ± 0.065		1.4		238.8 ± 6.0		0.45		0.44		164.8		85.3 ± 3.1	
Germ	Bare	0.158 ± 0.089	NS	1.5		208.2 ± 17.4	**	0.28		0.13		38.8		48.5 ± 1.7	**
	Seagrass	0.067 ± 0.004		1.5		246.7 ± 3.6		0.11		0.09		22.9		18.6 ± 1.6	
Font	Bare	0.020 ± 0.004	*	0.1		243.0 ± 10.6	NS	0.23		0.37		21.7		2.3 ± 2.2	**
	Seagrass	0.096 ± 0.022		0.8		240.5 ± 1.4		0.26		0.30		78.9		53.9 ± 4.4	
Garr	Bare	0.751 ± 0.090	NS	1.3		188.3 ± 7.8	NS	0		0		0		-13.0 ± 5.1	**
	Seagrass	0.646 ± 0.184		0.6		201.8 ± 3.6		0.32		0.33		92.2		78.9 ± 3.0	
Gail	Bare	0.164 ± 0.015	*	0.9		229.0 ± 2.2	NS	0		0		0		5.2 ± 1.8	**
	Seagrass	0.107 ± 0.019		2.0		209.3 ± 13.1		0.15		0.11		62.6		47.8 ± 1.7	
Ande	Bare	0.011 ± 0.003	**	0.5		250.7 ± 12.3	#	0.29		0.40		74.5		55.8 ± 5.3	**
	Seagrass	0.081 ± 0.029		1.1		242.8 ± 4.4		0.35		0.28		129.9		104.0 ± 1.6	
All	Bare	0.256 ± 0.047	NS	1.0 ± 0.1	NS	217.8 ± 5.0	NS	0.162 ± 0.019	**	0.192 ± 0.026	NS	33.3 ± 4.3	***	26.2 ± 4.5	***
	Seagrass	0.232 ± 0.042		1.1 ± 0.1		225.9 ± 3.7		0.247 ± 0.020		0.230 ± 0.021		80.6 ± 7.9		56.2 ± 5.3	

Supplementary figures

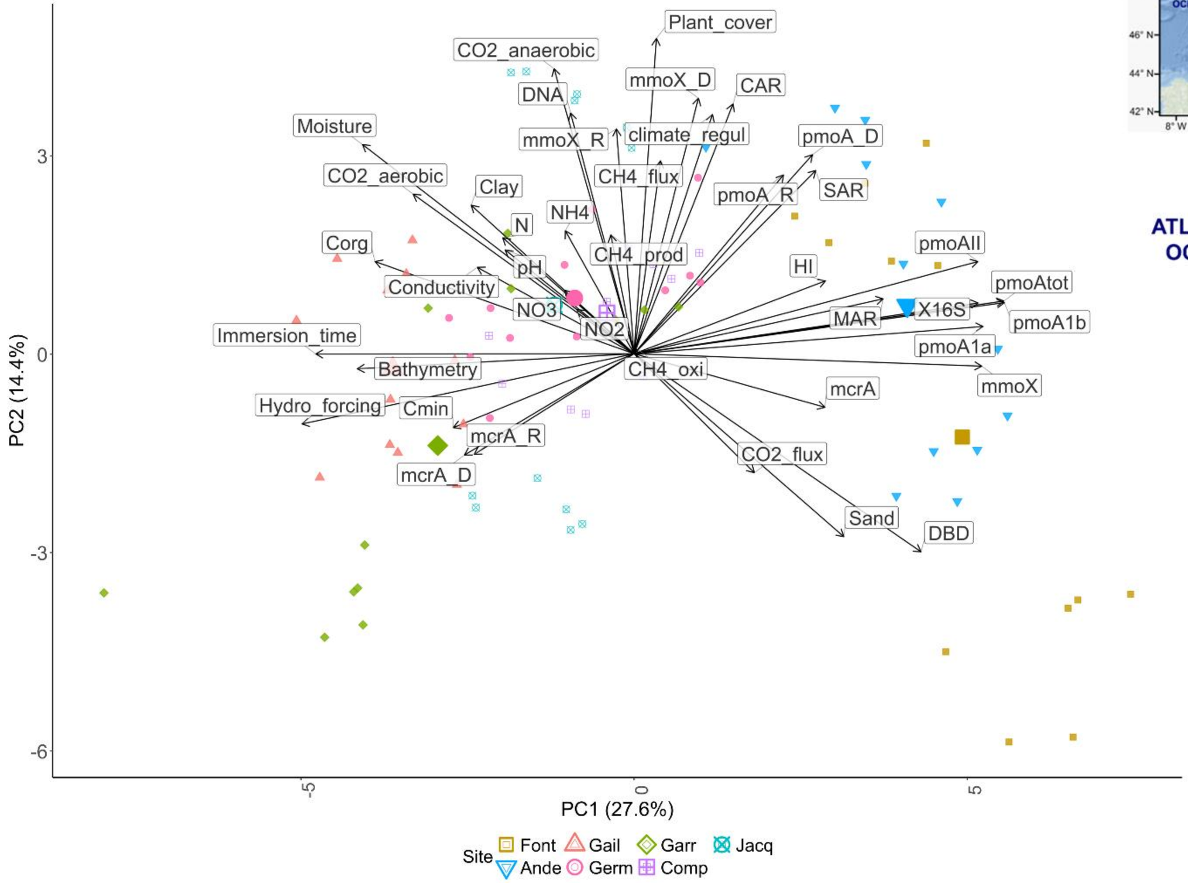

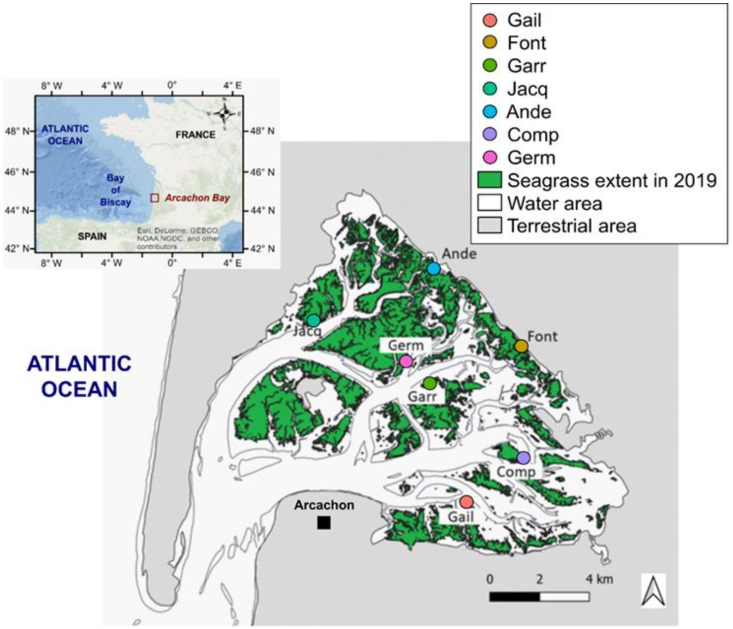

Figure S1 Principal Component Analysis (PCA) biplot illustrating the relationships among physical, chemical, microbial, and GHG variables across seven sites (n = 83 samples), accompanied by a map of Arcachon Bay. Arrows indicate the contribution of each variable to the ordination. Both axes were rescaled (×6) to improve visualization of sites and variables. Sites are represented by different shapes and colors, with larger symbols denoting the barycenter of each site. The map uses the same color scheme for each site as the PCA plot, facilitating comparison. This supplementary figure confirms the ordination pattern shown in Figure 2 of the main text. Together, the PCA and map highlight the PC1 gradient, which reflects a large-scale ecological gradient linked to ocean connectivity and sediment stability. Sites closer to the ocean inlet, located on the left side of both figures, are characterized by longer immersion times, stronger current flows, and greater depths (higher bathymetry), such as Gail and Garr. In contrast, the sites Ande and Font, positioned on the right side of PC1, stand out as distinct and more isolated. These sites lie in the inner part of the bay, indicating a lower degree of connection to the open ocean.

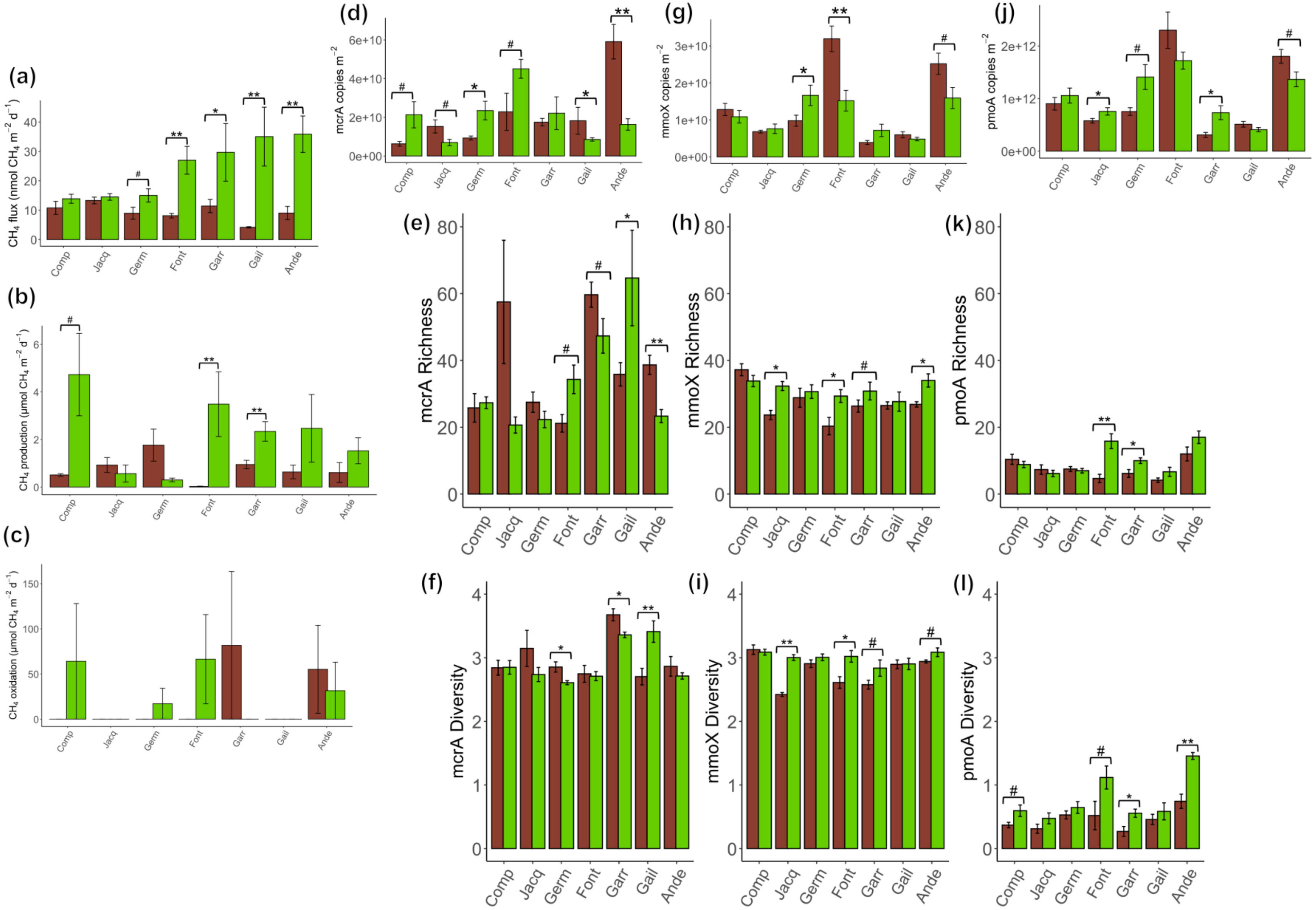

(a)
CH4 flux (nmol CH4 m-2 d-1)
(b)
CH4 production (µmol CH4 m-2 d-1)
(c)
CH4 oxidation (µmol CH4 m-2 d-1)
(d)
mcrA copies m-2
(e)
mcrA Richness
(f)
mcrA Diversity
(g)
mmoX copies m-2
(h)
mmoX Richness
(i)
mmoX Diversity
(j)
pmoA copies m-2
(k)
pmoA Richness
(l)
pmoA Diversity
Comp
Jacq
Germ
Font
Garr
Gail
Ande

Figure S2 Comparison of CH_4 fluxes, CH_4-related processes, and microbial gene features across sites and sediment types in Arcachon Bay. Panels a-c show measured CH_4 flux (a), potential CH_4 production (b), and CH_4 oxidation rates (c) across sites. Panels d-f display *mcrA* gene copy numbers (d), richness (e), and diversity (f). Panels g-i show equivalent metrics for *mmoX* gene (g) copy number, (h) richness, (i) diversity. Panels j-l show *pmoA* gene copy numbers (j), richness (k), and diversity (l). Each bar represents mean ± standard error (n = 6 per subsite; n = 5 for Comp-Bare). Colors indicate sediment type: green = seagrass, brown = bare. Significant differences (Wilcoxon tests) between bare and vegetated sediments are indicated as follows: # ($p < 0.1$), * ($p < 0.05$), ** ($p < 0.01$).

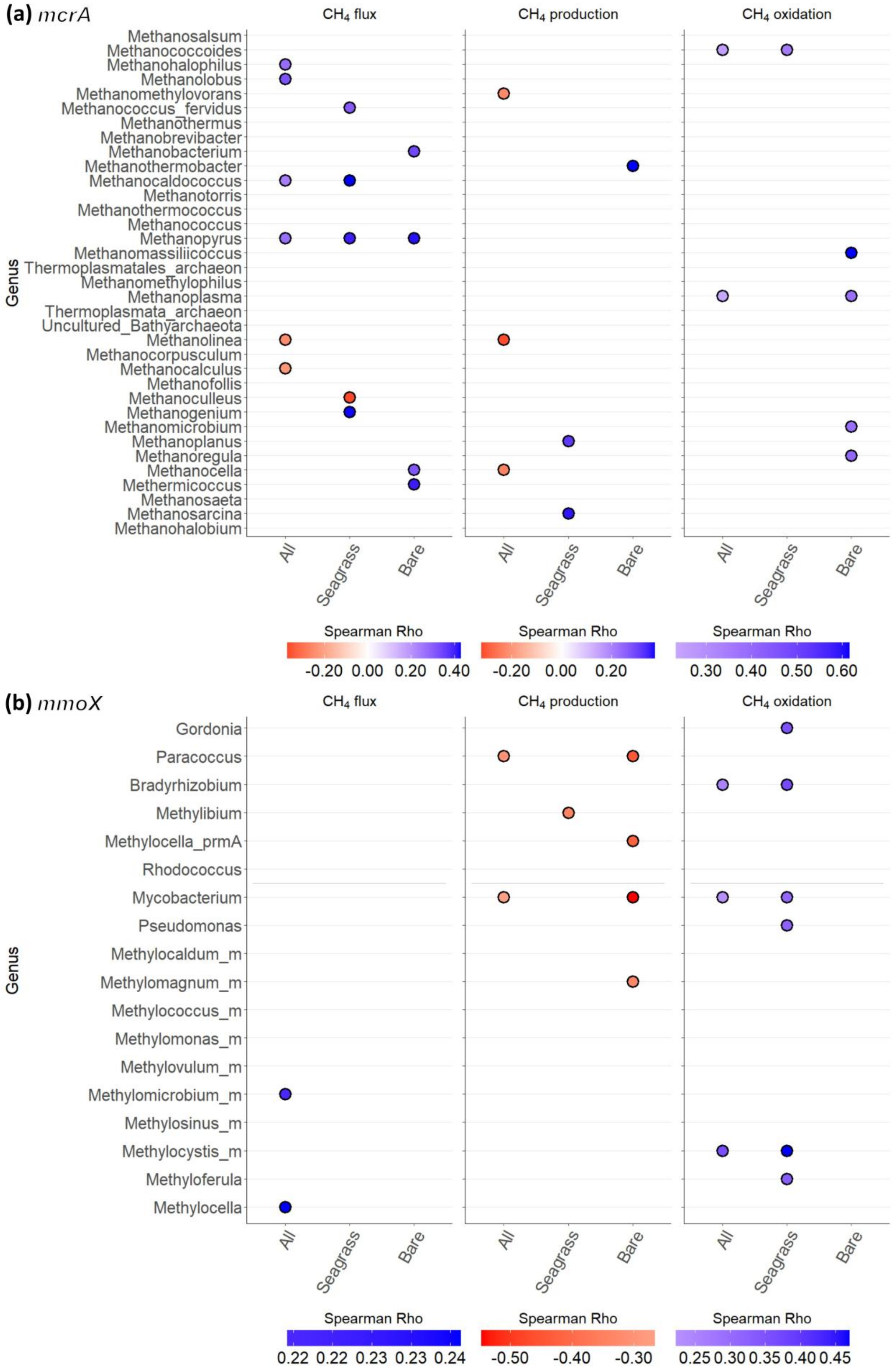
(a) mcrA
CH4 flux
CH4 production
CH4 oxidation
Genus
Methanosalsum
Methanococcoides
Methanohalophilus
Methanolobus
Methanomethylovorans
Methanococcus_fervidus
Methanothermus
Methanobrevibacter
Methanobacterium
Methanothermobacter
Methanocaldococcus
Methanotorris
Methanothermococcus
Methanococcus
Methanopyrus
Methanomassiliicoccus
Thermoplasmatales_archaeon
Methanomethylophilus
Methanoplasma
Thermoplasmata_archaeon
Uncultured_Bathyarchaeota
Methanolinea
Methanocorpusculum
Methanocalculus
Methanofollis
Methanoculleus
Methanogenium
Methanomicrobium
Methanoplanus
Methanoregula
Methanocella
Methermicoccus
Methanosaeta
Methanosarcina
Methanohalobium
All
Seagrass
Bare
Spearman Rho
-0.20 0.00 0.20 0.40
Spearman Rho
-0.20 0.00 0.20
Spearman Rho
0.30 0.40 0.50 0.60
(b) mmoX
CH4 flux
CH4 production
CH4 oxidation
Genus
Gordonia
Paracoccus
Bradyrhizobium
Methylibium
Methylocella_prmA
Rhodococcus
Mycobacterium
Pseudomonas
Methylocaldum_m
Methylomagnum_m
Methylococcus_m
Methylomonas_m
Methylovulum_m
Methylomicrobium_m
Methylosinus_m
Methylocystis_m
Methyloferula
Methylocella
All
Seagrass
Bare
Spearman Rho
0.22 0.22 0.23 0.23 0.24
Spearman Rho
-0.50 -0.40 -0.30
Spearman Rho
0.25 0.30 0.35 0.40 0.45

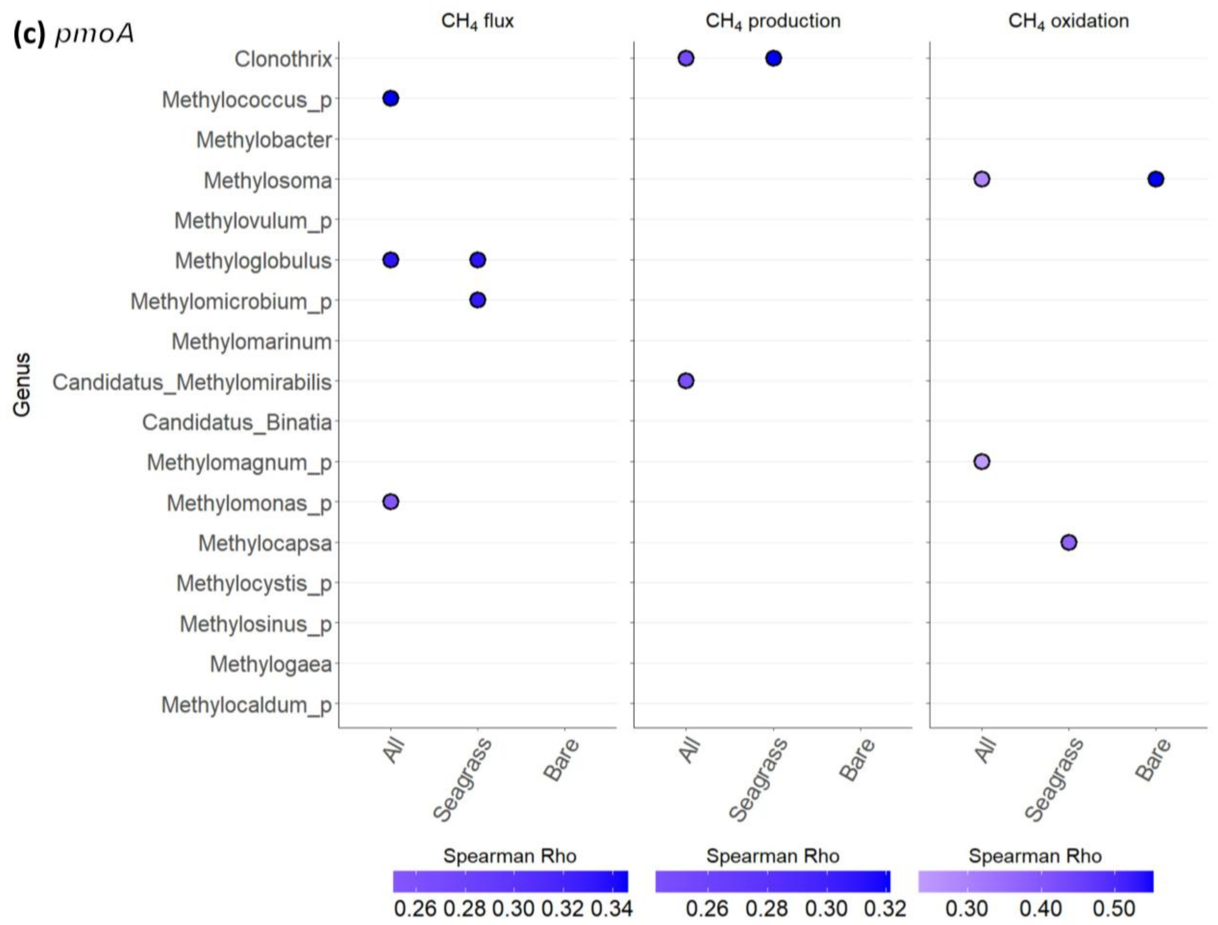

Figure S3 Significant Spearman correlations (Rho) between genus abundances and CH_4-related gases. Panel (a) shows genera identified from the *mcrA* gene (a methanogenesis marker), panel (b) from *mmoX*, and panel (c) from *pmoA* (both methanotrophy markers). For each gene, the first column lists genera significantly correlated with CH_4 flux, the second column with CH_4 production, and the third with CH_4 oxidation. Colors indicate the strength and direction of the correlation (blue = positive, red = negative), n = 83.

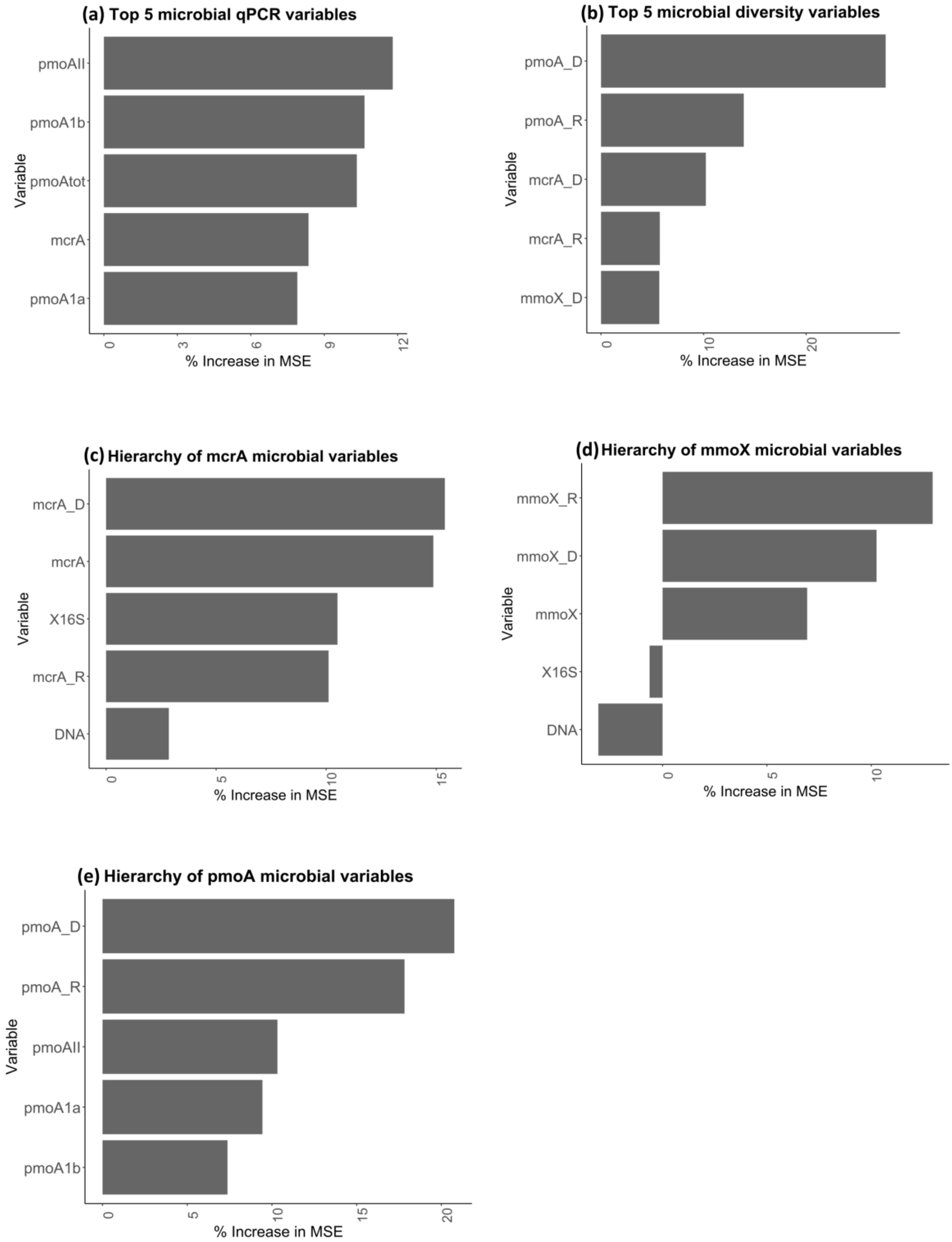
(a) Top 5 microbial qPCR variables
pmoAII
pmoA1b
pmoAtot
mcrA
pmoA1a
Variable
0
3
6
9
12
% Increase in MSE
(b) Top 5 microbial diversity variables
pmoA_D
pmoA_R
mcrA_D
mcrA_R
mmoX_D
Variable
0
10
20
% Increase in MSE
(c) Hierarchy of mcrA microbial variables
mcrA_D
mcrA
X16S
mcrA_R
DNA
Variable
0
5
10
15
% Increase in MSE
(d) Hierarchy of mmoX microbial variables
mmoX_R
mmoX_D
mmoX
X16S
DNA
Variable
0
5
10
% Increase in MSE
(e) Hierarchy of pmoA microbial variables
pmoA_D
pmoA_R
pmoAII
pmoA1a
pmoA1b
Variable
0
5
10
15
20
% Increase in MSE

Figure S4 Variable importance in random forest analysis identifying key microbial predictors of methane (CH_4) fluxes. Importance was assessed using the mean increase in mean squared error (% Increase in MSE) after permutation of each variable, n = 83. (a-b) Top 5 predictors within each microbial category: (a) qPCR-based variables, (b) diversity indices. (c-e) Hierarchical importance of microbial predictors associated with the *mcrA*, *mmoX*, and *pmoA* genes, respectively.
